# Multifunctional Dy^3+^ Complexes with Triphenylmethanolates: Structural Diversity, Luminescence, and Magnetic Relaxation

**DOI:** 10.3390/molecules29225343

**Published:** 2024-11-13

**Authors:** Gautier Félix, Aleksei O. Tolpygin, Aurore Larquey, Ilia A. Gogolev, Yulia V. Nelyubina, Yannick Guari, Joulia Larionova, Alexander A. Trifonov

**Affiliations:** 1 ICGM, University Montpellier, CNRS, ENSCM, 34293 Montpellier, France; aurore.larquey@umontpellier.fr (A.L.); yannick.guari@umontpellier.fr (Y.G.); 2 G.A. Razuvaev Institute of Organometallic Chemistry of Russian Academy of Sciences, 49 Tropinina Str., GSP-445, 603950 Nizhny Novgorod, Russia; toao2@yandex.ru; 3 A.N. Nesmeyanov Institute of Organoelement Compounds of Russian Academy of Sciences, 28 Vavilova Str., bld. 1, 119334 Moscow, Russia; illiago135@gmail.com (I.A.G.); unelya@ineos.ac.ru (Y.V.N.)

**Keywords:** Single Molecule Magnets, Dysprosium, magnetic relaxation, luminescence, lanthanides

## Abstract

The coordination environment of magneto-luminescent Dy^3+^-based Single-Molecule Magnets (SMM) is a crucial factor influencing both magnetic and luminescent properties. In this work, we explore how triphenylmethanolate (Ph_3_CO^−^), in combination with other ligands, can modulate the structure and, therefore, the magnetic properties of Dy^3+^-based SMM. Using triphenylmethanolate in combination with THF and pyridine (Py) as co-ligands, we synthesized a series of mononuclear *cis*-[Dy(OCPh_3_)_2_(THF)_4_][BPh_4_]·(2,6-Me_2_C_5_H_3_N) (**1**), *trans*-Dy(OCPh_3_)_3_(THF)_2_ (**2**), *fac*-Dy(OCPh_3_)_3_(py)_3_ (**3**) and dinuclear [(Ph_3_CO)Dy(THF){(μ^2^–Cl)_2_Li(THF)_2_}μ^2^–Cl]_2_ (**4**) complexes where the Dy^3+^ ion presents five- or six-coordinate geometries. Dinuclear compound **4** exhibits a genuine SMM behavior with a relatively high energy barrier of 421 cm^−1^, while mononuclear complexes **1**–**3** are field-induced SMM. These complexes also present Dy^3+^-characteristic luminescence, highlighting their multifunctional character.

## 1. Introduction

Lanthanide ion-based Single Molecule Magnets (SMMs) have garnered significant attention over recent decades, particularly since the discovery of the mononuclear dysprosium metallocene family, which exhibits magnetic behavior at temperatures approaching the boiling point of liquid nitrogen [1,2,3,4]. This breakthrough has opened exciting possibilities for utilizing SMMs in various applications that aim for miniaturization, including spintronic devices, quantum computing, and high-density data storage. The unique magnetic properties of these molecules make them promising candidates for next-generation technologies where control at the molecular level is crucial [5,6,7]. The high magnetic anisotropy and large magnetic moments observed in certain Ln^3+^-based SMMs are critical factors enabling their slow magnetic relaxation and molecular-level hysteresis. Among the various lanthanide ions, the Kramers ion Dy^3+^, with its doubly degenerate ground state and oblate-shaped electronic density, forms a promising magnetic ground state with a well-isolated first excited state, reassembling the essential factors for achieving strong magnetic anisotropy [8,9,10]. For this reason, it stands as a prime candidate for the development of high-performance SMMs. Numerous examples of mono- and polynuclear Dy^3+^-based SMMs have been reported in the literature with large energy barriers and elevated blocking temperatures [11,12,13,14,15,16]. The key strategy in developing high-performing SMMs consists of designing complexes where the magnetic axiality may be achieved with a strong axial field even without strict point symmetry [9]. In this connection, significant effort has been dedicated to understanding how the coordination environment of the Dy^3+^ ion influences its magnetic properties. A textbook example of such magneto–structural correlation, which led to groundbreaking advancements in the field of SMM research, can be found in the renowned family of sandwich-like mononuclear Dy^3+^ metallocene compounds, [(Cp^R^)_2_Dy]^+^ (where Cp = cyclopentadienyl anion and R = Cp substituent group) [1,2,3,4]. It has been demonstrated that improving the linearity of the Cp_centroid_–Dy–Cp_centroid_ angles, along with shortening the Dy–C(Cp) bonds, significantly enhances SMM performance. Notably, the compound [(Cp*)Dy(Cp^iPr^_5_)][B(C_6_F_5_)_4_], with a blocking temperature of 80 K, set a remarkable milestone by exceeding the boiling point of liquid nitrogen. In this series, isopropyl substituents in [(Cp*)Dy(Cp^iPr^_5_)][B(C_6_F_5_)_4_] promote a wide Cp_centroid_–Dy–Cp_centroid_ angle of 162.5° and short Cp_centroid_–Dy distances of 2.2961(1) and 2.284(1) Å, while also providing sufficient steric hindrance to prevent the coordination of equatorial ligands [2]. Beyond the sandwich-like mononuclear Dy^3+^ metallocenes, other families of mononuclear Dy^3+^-based SMM aiming to achieve high magnetic axiality and explore its influence on magnetic relaxation mechanisms have been investigated. Notably, a strategy involving the design of mononuclear 7–or 6-coordinated complexes by positioning two strongly coordinating ligands (such as alkoxide, aryloxide, silanolate, halogen, etc.) in the axial positions to the Dy^3+^ ion with less coordinating ligands (THF, pyridine, methylpyridine) in the equatorial ones has shown promise in enhancing magnetic performance [11,13,17,18,19,20,21,22].

In addition to the optimization of magnetic relaxation behavior in SMM, significant research has been conducted on the combination of SMM behavior with luminescence. The objective is to develop multifunctional magneto-luminescent complexes in which the optical properties are often correlated with effective energy barriers from dynamic magnetic measurements. This approach facilitates the elucidation of magnetic relaxation mechanisms [23,24,25,26]. A review of the literature reveals that Dy^3+^-based complexes are the most commonly explored for the design of magneto-luminescent SMMs, primarily due to the lanthanide’s ability to emit in the visible region. However, it is widely acknowledged that Dy^3+^ complexes are less luminescent compared to their Eu^3+^ and Tb^3+^ counterparts, which typically exhibit a higher quantum yield visible emission [26]. However, the development of efficient luminescent SMMs remains a significant challenge, as the criteria for enhancing magnetic axiality and optimizing the “antenna effect” to boost the relatively weak lanthanide luminescence often conflict. This creates a delicate balancing act in selecting appropriate ligands and designing the coordination environment to satisfy both objectives. It is not surprising that most reported cases utilize well-known “antenna” ligands, such as β-diketonates and Schiff bases, which form complexes with high coordination numbers. However, this makes it challenging to design highly axial complexes, often resulting in limited SMM performance. To the best of our knowledge, the luminescent properties of aryloxide or alkoxide SMM have been investigated for these SMMs only scarcely. In this context, we successfully employed triphenylalkoxide ligand to design a 6-coordinated *trans*-[Dy(OCPh_3_)_2_(THF)_4_][BPh_4_] complex, which demonstrated a high energy barrier of 1385 cm^−1^ and a Dy^3+^ characteristic luminescence [27]. In this compound, octahedral geometry promotes the high blocking barrier based on the quenching of one-phonon transitions between the three low-lying crystal-field multiplets due to large energy gaps between them exceeding the available phonon energies and forcing the activated relaxation to proceed through the fourth doublet.

In this article, we investigate a series of magneto-luminescent Dy^3+^-based SMM by utilizing strongly coordinating triphenylalkoxide (Ph_3_CO^−^) ligands in combination with weakly-coordinating THF and even less coordinating pyridine co-ligands to demonstrate the impact of the Dy^3+^ ion geometry on their physical properties. We synthesized a series of mononuclear cis-[Dy(OCPh_3_)_2_(THF)_4_][BPh_4_]·(2,6-Me_2_C_5_H_3_N) (**1**), *trans*-Dy(OCPh_3_)_3_(THF)_2_ (**2**), *fac*-Dy(OCPh_3_)_3_(py)_3_ (**3**) and dinuclear [(Ph_3_CO)Dy(THF){(μ^2^-Cl)_2_Li(THF)_2_}μ^2^-Cl]_2_ (**4**) complexes featuring five- and six-coordinated Dy^3+^ ion. Dinuclear compound **4** exhibits a genuine SMM behavior with a relatively high energy barrier of 421 cm^−1^, while mononuclear complexes **1**–**3** are field-induced SMM. They also present Dy^3+^-characteristic luminescence, highlighting their multifunctional character.

## 2. Results and Discussion

### 2.1. Synthesis and Structures

**Scheme 1:**
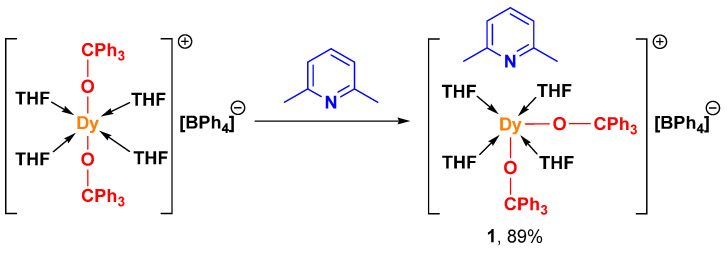
Synthesis of complex **1**.

The treatment of *trans-*[Dy(OCPh_3_)_2_(THF)_4_][BPh_4_] [27] with 2,6-dimethylpyridine (24 h, ambient T) and the subsequent recrystallization of the reaction product from 2,6-Me_2_C_5_H_3_N/hexane mixture did not allow for a substitution of THF but afforded a solvate *cis*-[Dy(OCPh_3_)_2_(THF)_4_][BPh_4_]·(2,6-Me_2_C_5_H_3_N) (**1**) (Scheme 1). Complexes **1** was isolated in 89% yield as a solvate with one lutidine molecule *cis*-[Dy(OCPh_3_)_2_(THF)_4_][BPh_4_]·(2,6-Me_2_C_5_H_3_N). Colorless crystals suitable for single crystals X-ray diffraction investigations are readily soluble in THF but almost insoluble in hexane.

**Scheme 2:**
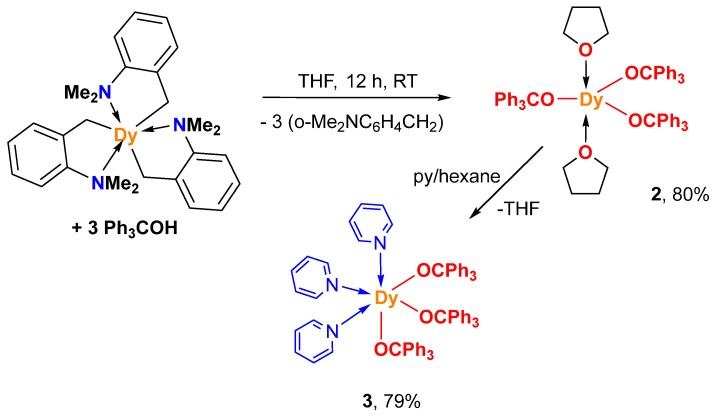
Synthesis of complexes **2** and **3**.

In order to synthesize a tris(alkoxide) Dy^3+^ complex containing Ph_3_CO^−^ ligands, an alkane elimination approach consisting of the reaction of Dy(o-Me_2_NC_6_H_4_CH_2_)_3_ with three equivalents of Ph_3_COH was used (Scheme 2). After recrystallization of the reaction product from the THF/hexane mixture, the complex Dy(OCPh_3_)_3_(THF)_2_ (**2**) was isolated as colorless crystals in an 80% yield. Recrystallization of **2** from pyridine/hexane mixture resulted in colorless crystals of *fac*-Dy(OCPh_3_)_3_(py)_3_ (**3**) in 79% yield.

**Scheme 3:**
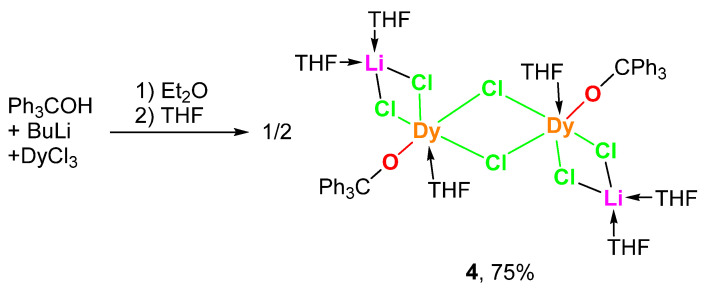
Synthesis of complex **4**.

For the synthesis of complex **4,** a different synthetic approach was employed. A salt metathesis reaction of lithium alkoxide [Ph_3_COLi] in situ generated from Ph_3_COH and *n*-BuLi (Et_2_O, 0 °C) with equimolar amount of anhydrous DyCl_3_ in THF at 65 °C (80 h) afforded **4** in 75% yield (Scheme 3). Complex **4** was isolated as colorless crystals after recrystallization of the reaction product from the THF/hexane mixture at room temperature.

Single crystal X-ray diffraction studies revealed that complexes **1** and **2** crystallize in the monoclinic space group *P*2_1_/*n*, while **3**, in the trigonal *R*3 space group with a unique dysprosium site (Table S1, Electronic Supporting Information (ESI)). The dinuclear complex **4** crystallizes in the triclinic space group *P-1*, with half of the molecule being symmetry-independent.

**Figure 1:**
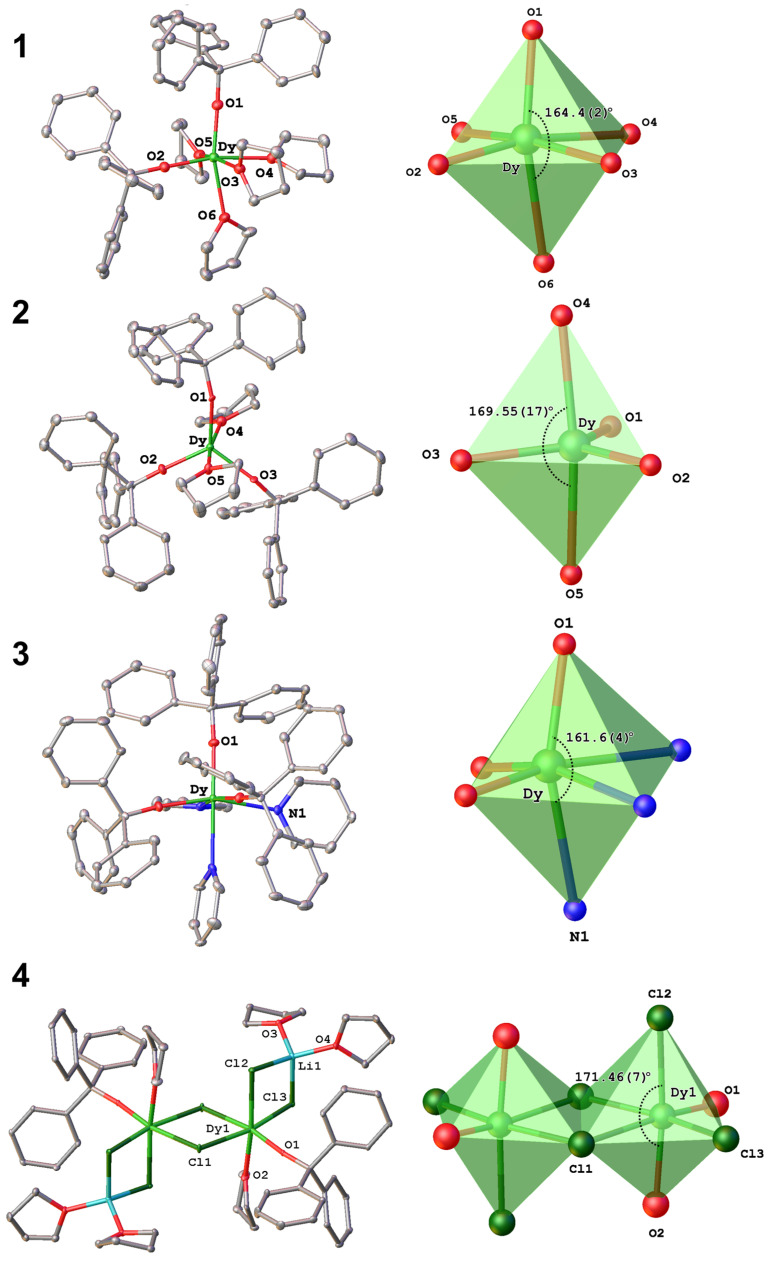
General views of the molecular structures of **1**–**4** showing the coordination environment of Dy^3+^ ions (**left**) and the corresponding coordination polyhedra (**right**). Hydrogen atoms and minor components of the disordered ligands are omitted, atoms are shown as thermal ellipsoids (*p* = 20%), and only symmetry-independent heteroatoms are labeled.

Notably, in complex **1**, 2,6-dimethylpyridine did not displace the THF molecules from the equatorial plane of the Dy^3+^ ion of the precursor *trans-*[Dy(OCPh_3_)_2_(THF)_4_][BPh_4_]. However, its presence led to a significant change in the arrangement of the ^−^OCPh_3_ ligands, shifting from a strongly axial configuration (O–Dy–O 180°) in the parent complex to a *cis* configuration (O–Dy–O 102.7(2)°) in **1** (Figure 1). Moreover, in comparison with the parent *trans-*[Dy(OCPh_3_)_2_(THF)_4_][BPh_4_] compound having the same coordination number and coordination environment of the Dy^3+^ ion, the Dy–OCPh_3_ bonds in the solvate **1** are noticeably shorter (2.062(5) and 2.054(5) Å vs. 2.103(1) Å). At the same time, an extension of the Dy–O_THF_ bond lengths in **1** (2.341(5)–2.449(6) Å) was observed (for comparison, Dy–O_THF_ in [Dy(OCPh_3_)_2_(THF)_4_][BPh_4_] are of 2.370(1) and 2.398(1) Å). In **1**, the cationic dysprosium complex incorporates two alkoxide ligands in the *cis* position, whereas four THF molecules complete the coordination sphere to give a six-coordinated complex with a slightly distorted octahedral geometry (Table 1). Although not isostructural, the structure is reminiscent of the silanolate and stannanolate complexes we reported recently [22]. The shortest Dy–O distances involving the alkoxide ligands are equal to 2.054(5) and 2.062(5) Å with an O2–Dy–O3 angle of 102.7(2)°. In comparison, the Dy–O^−^ bonds in the *trans*-[Dy(OCPh_3_)_2_(THF)_4_][BPh_4_] complex are slightly longer (2.103(1) Å) [27]. On the other hand, the observed Dy–O^−^ bonds in **1** are shorter than those found in *cis*-[Dy(OSiPh_3_)_2_(THF)_4_][BPh_4_] (2.093(5)/2.108(5) Å) and comparable to those of *cis*-[Dy(O–nPh_3_)_2_(THF)_4_][BPh_4_] (2.068(3) Å) complexes. As expected, the Dy–O_THF_ bonds in **1** are significantly longer, with the values ranging between 2.341(5) and 2.449(6) Å.

In compound **2**, the Dy^3+^ ion is five-coordinated and adopts a slightly distorted trigonal bipyramidal geometry (Figure 1). Three oxygen atoms of Ph_3_CO^−^ ligands are located in the equatorial plane around the Dy^3+^ ion, while the axial positions of the bipyramid are occupied by two THF molecules (O_THF_–Dy–O_THF_ angle is 169.57(16)°). The Dy–O_Ph3CO_ bond lengths (1.980(17)–2.102(5) Å), as well as the Dy–O_THF_ bond lengths (2.362(5), 2.407(5) Å) (Table 1) in **2** correspond to those of the related five-coordinated tris(aryloxide) complex Dy(2,6-^i^Pr_2_C_6_H_3_O)_3_(THF)_2_ [28]. The ^−^O–Dy–O^−^ angles range between 97.8(8) and 163.0(10)° while the O_THF_–Dy–O_THF_ angle gently deviates from linearity with a value of 169.57(16)°.

Recrystallization of five-coordinated compound **2** from pyridine–hexane mixture afforded the six-coordinated complex **3** featuring an octahedral coordination environment of the Dy^3+^ ion (Figure 1). Notably, the substitution of THF by pyridine led not only to an increase in the coordination number but also to a change in the mutual arrangement of the Ph_3_CO^−^ ligands. In **3**, three Ph_3_CO– ligands occupy *fac*-positions of an octahedron. The Dy–O^−^ bond lengths (2.124(8) Å) and the Dy–N_Pyr_ bond lengths (2.601(11) Å) in **3** are in the range of values typical for previously described tris(alkoxide) dysprosium complexes featuring octahedral geometry (Dy(OC_14_H_9_)_3_(py)_3_ Dy–O 2.125-2.127 Å, Dy–N 2.603-2.605 Å [29]; Dy(2,6-Me_2_C_6_H_3_O)_3_(py)_3_ Dy–O 2.094-2.118 Å, Dy-N 2.521-2.556 Å; Dy(2,6-^i^Pr_2_C_6_H_3_O)_3_(py)_3_ Dy–O 2.116-2.118 Å, Dy–N 2.572-2.615 Å) [28].

Finally, compound **4** is a neutral six-coordinated complex incorporating three triphenylalkoxide and three pyridine ligands defining a *fac* octahedral isomer. The space group *R*3 imposes that there are only one Dy–O and one Dy–N distances of 2.124(8) and 2.601(11) Å, respectively, whereas the ^−^O–Dy–O^−^ angle is equal to 102.4(3)°. While all the above-described complexes have a unique dysprosium site, **4** is a centrosymmetric heterobimetallic dimer. Two (Ph_3_CO)(THF)Dy fragments are linked by two μ^2^–Cl ligands, thus forming a perfectly planar, rhombic fragment Dy_2_Cl_2_ with the Dy–Dy distance of 4.1754(7) Å. Each Dy^3+^ ion is linked by two μ^2^–Cl ligands with Li(THF)_2_ moiety, resulting in a tetranuclear core. Thereby, the coordination polyhedron of the six-coordinated Dy^3+^ ion adopts a slightly distorted octahedral geometry. The only Dy–O_Ph3CO_ bond (2.0335(12) Å) in **4** is noticeably shorter than in the related octahedral complexes [Dy(OCPh_3_)_2_(THF)_4_][BPh_4_] (2.103(1) Å) [19], [Dy(OtBu)_2_(4-py-Ph)_4_][BPh_4_] (2.066(8) Å) [27], [Dy(OC(CF_3_)_3_)_2_(THF)_4_][BPh_4_] (2.11(4)-2.15(3) Å) [30]. The Dy–O_THF_ bond (2.3473(15) Å) is much longer; however, its length agrees with those in the complexes featuring similar geometry of the coordination environment of Dy^3+^ ion [19,27]. The lengths of μ^2^-bridging Dy–Cl bonds, which fall into the rather broad range of 2.6856(4)–2.7694(4) Å, are also as expected for six-coordinate Dy complexes [31]. One of the O_THF_–Dy–Cl (171.46(7)/164.59(8)°) angles and one of the O_Ph3CO_–Dy–Cl angles (169.78(4)°) are close to linear while others (O_THF_–Dy–Cl 82.21(4) –92.16(6)°; O_Ph3CO_–Dy–Cl 93.38(4)–103.34(7)°) do not deviate much from 90°; the O–Dy–O angle is 89.70(5)°.

The crystal packings for compounds **1**–**4** are shown in Figure S1 (ESI). In all cases, the shortest Dy…Dy intermolecular distances are below 13 Å and equal to 12.664(5), 11.1584(8), 10.4932(10), and 10.0466(2) Å for **1**, **2**, **3,** and **4,** respectively.

### 2.2. Magnetic Properties

The magnetic properties of all compounds were investigated by using a SQUID MPMS3 magnetometer from Quantum Design International (USA), working between 1.8–350 K up to 7 T.

**Figure 2:**
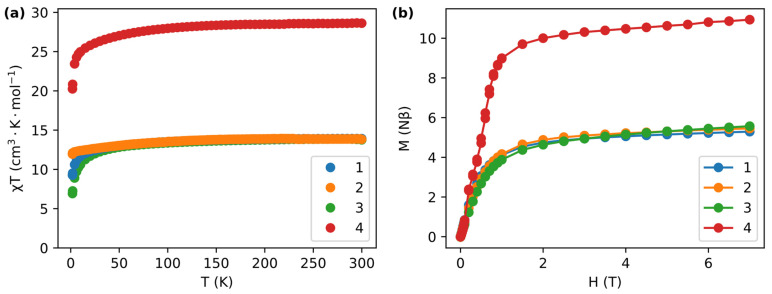
(**a**) χT vs. temperature curves performed with an applied static magnetic field of 1000 Oe for samples **1**–**4**; (**b**) First magnetization curves as a function of the external magnetic field performed at 1.8 K for **1**–**4**.

**Figure 3:**
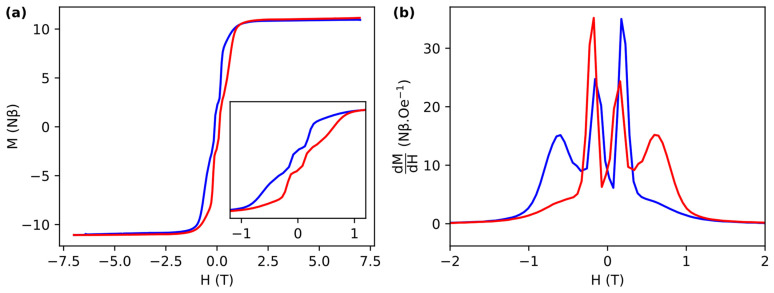
(**a**) Hysteresis loop performed at 1.8 K with an absolute value of the magnetic field speed rate of 100 Oe.s^−1^ for samples **4**. Inset: magnification of the hysteresis loop between −1.2 and 1.2 T. (**b**) Derivative of the magnetization with respect to the magnetic field as a function of the external magnetic field. The blue and red lines represent the evolution of the magnetization when the field speed rate is −100 Oe.s^−1^ and +100 Oe.s^−1^, respectively.

Figure 2a shows the temperature dependence of the *χT* product performed with the static applied magnetic field of 1000 Oe for samples **1**–**4**. The *χT* values obtained for samples **1**–**3** at 300 K are equal to 13.9 cm^3^.K.mol^−1^, which is close to the theoretical value of 14.17 cm^3^.K.mol^−1^ expected at high temperature for Dy^3+^ ion in the free-ion approximation (*J* = 15/2, *g* = 4/3) [32]. As the temperature decreases, the *χT* value decreases starting from around 30 K, which indicates the thermal depopulation of the *m_J_* levels. The presence of possible antiferromagnetic interactions between adjacent Dy^3+^ ions cannot be excluded since intermolecular distances are not very large. The maximum *χT* value obtained at 300 for sample **4** is 28.6 cm^3^.K.mol^−1^, which is close to the high-temperature theoretical value calculated for two Dy^3+^ ions non-implicated in magnetic interaction (28.34 cm^3^.K.mol^−1^). Figure 2b shows the magnetization curves as a function of the external magnetic field performed at 1.8 K. The maximum values obtained at 7 Tesla are 5.46 Nβ, 5.57 Nβ, and 5.29 Nβ for samples **1**, **2**, and **3**, respectively. These values are lower compared to the expected theoretical value, which is 10.0 Nβ. However, a strong axial magnetic anisotropy can lead to a decrease in the magnetization saturation value around 5.0 Nβ [33]. The higher experimental values can be attributed to a minor mechanical movement of the magnetic sample powder, which exhibits a tendency to align with the external continuous magnetic field. No hysteretic behavior was observed for these compounds. With regard to sample **4**, the maximum value obtained at 7 Tesla is 11.0 Nβ, which is comparable to the previously observed values for Dy^3+^-based dinuclear compounds with strong axial anisotropy [34]. The hysteresis loop, displayed in Figure 3a, is open at 1.8 K with a modest coercive field of 3200 Oe, which is consistent with the blocking of the magnetization in SMM. There are three steps on the hysteresis loop (at −1610, 0 and 1610 Oe) (Figure 3b), which are indicative of the ground state (zero applied static filed) and resonant Quantum Tunneling of the Magnetization (QTM).

**Figure 4:**
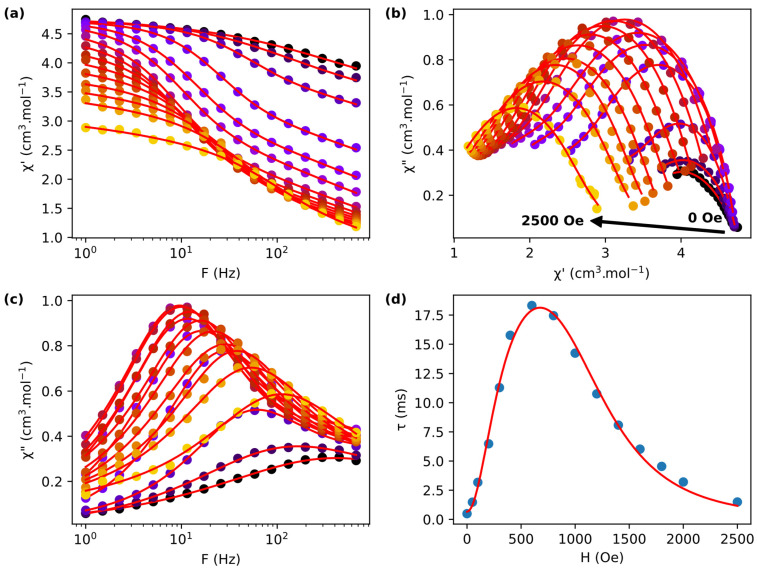
Frequency dependence of χ’ (**a**) and χ” (**c**) for **1** at 1.8 K performed under various applied DC fields. (**b**) Cole–Cole plots obtained using the frequency dependence of χ” for **1** at 1.8 K under various DC fields. The color gradient, which ranges from dark violet to yellow, represents the variation in external magnetic field intensity, with values ranging from 0 to 2500 Oe, respectively. The solid lines correspond to the best fit obtained with a generalized Debye model. (**d**) Field dependence of the relaxation time for **1**. The red line represents the fit using Equation (1).

The dynamic magnetic properties of mononuclear compounds **1**–**3** were investigated in alternating current (AC) mode to examine the occurrence of slow magnetic relaxation. Since all of them present comparable field-induced SMM behavior, the description is given only for **1**. The in-phase, *χ*’, and the out-of-phase, *χ*”, components of the AC susceptibility for **1** present a zero signal in a zero-static field, which can be explained by the presence of the fast QTM. To avoid its influence, the frequency dependence of the AC susceptibility was measured under different applied continuous (DC) fields, which showed a strong signal for both components (Figure 4a,c). The corresponding Cole–Cole (Argand) plot fitted with a generalized Debye model can be seen in Figure 4b. The field dependence of the relaxation time (Figure 4d) was fitted with Equation (1) in order to determine the optimal magnetic field to suppress QTM:

(1) $$\tau^{-1}=A_{T}\cdot H^{4}+\frac{B_{1}}{1+B_{2}\cdot H^{2}}+C_{T}$$

where the first term accounts for the direct relaxation process for Kramers ions, with A_T_ serving as a constant at a given temperature. The second term represents the QTM process, with two constants, B_1_ and B_2_. The third term represents processes that are constant as a function of H at a given temperature, which could be Raman or Orbach processes. Note that in the case where B_2_⋅H^2^ >> 1, Equation (1) can be rewritten as follows:

(2) $$\tau^{-1}=A_{T}\cdot H^{4}+\frac{B}{H^{2}}+C_{T}$$

where the QTM process is approximated, and the constants B_1_ and B_2_ are replaced by a single constant, B.

**Figure 5:**
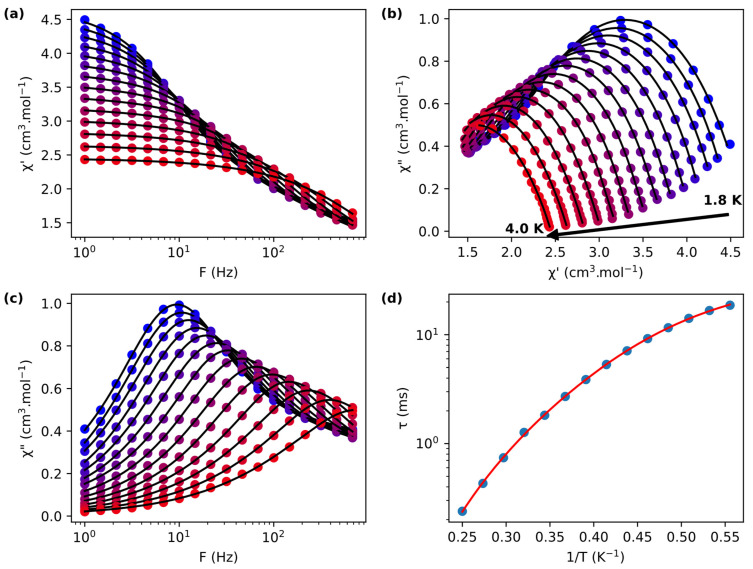
Frequency dependence of the in-phase, χ’, (**a**) and out-of-phase, χ” (**c**) components of the AC susceptibility for **1** under an optimal applied magnetic field of 750 Oe. The red lines are the result of the Cole–Cole fitting. (**b**) Cole–Cole plots obtained using the frequency dependence of χ” for **1** obtained under 750 Oe. The solid lines correspond to the best fit obtained with a generalized Debye model. The color gradient, which ranges from blue to red, represents the variation in temperature, with values ranging from 1.8 to 4.0 K, respectively. (**d**) Temperature dependence of the relaxation time for **1** (750 Oe) and the corresponding fit with Equation (3) (red solid line).

The maximum value of the relaxation time determines the optimal applied magnetic field. Therefore, the curve for **1** was fitted with Equation (1), which gave the optimal magnetic field of 750 Oe. This value was chosen, therefore, for further experiments for this sample. The frequency dependence of the in-phase and the out-of-phase components of the AC susceptibility performed under this optimal applied field of 750 Oe is shown in Figure 5a,b. It indicates the presence of a single frequency-dependent peak for *χ*”, which shifts towards higher frequency as the temperature increases (from 1.8 to 4 K). The corresponding Cole-Cole plot fitted with a generalized Debye model gave moderate *α* parameter values (between 0.1 and 0.25), indicating a certain distribution of the relaxation times (Figure 5b). The temperature dependence of the relaxation time was fitted by using Equation (3):

(3) $$\tau^{-1}=A_{H}\cdot T+\tau_{0}^{-1}\cdot \exp\left(-\frac{E}{k_{B}\cdot T}\right)+C\cdot T^{9}$$

where the first term represents the direct process, with A_H_ serving as a constant at a given external magnetic field. The second term represents the Orbach process, where *E* is the effective barrier energy. The third term represents the Raman process for Kramers ions, where *C* is a constant. Temperature dependence of the relaxation time was fitted with Equation (3) (Figure 5d), providing the best-fit parameters: *E* = 11.1 ± 0.4 cm^−1^, *τ*_0_ = (1.3 ± 0.3)× 10^−5^ s, C = (1.03 ± 0.07)× 10^−2^ and *A_H_* = 22 ± 1. These parameters indicate the presence of the direct and the Orbach processes in **1**, while Raman relaxation is rather weak. Note that in comparison to other previously cited mononuclear Dy^3+^-based SMMs with octahedral geometry, the energy barrier is rather modest, which can be explained by an important deviation from the linearity of the O–Dy–O angle (164.39°).

The dynamic behavior of compounds **2** and **3**, which present distorted pentagonal bipyramidal and octahedral geometries at the Dy site, respectively, was investigated in a similar manner. The results are shown in Figures S2–S5, ESI, and Table 2. These compounds present field-induced SMM behaviors with comparable energy barriers of 16.5 and 9.4 cm^−1^. Note that in the case of the 5-coordinated compound **2**, the Orbach relaxation is rather dominant, while for 6-coordinated complex **3**, the direct process represents the main relaxation.

**Figure 6:**
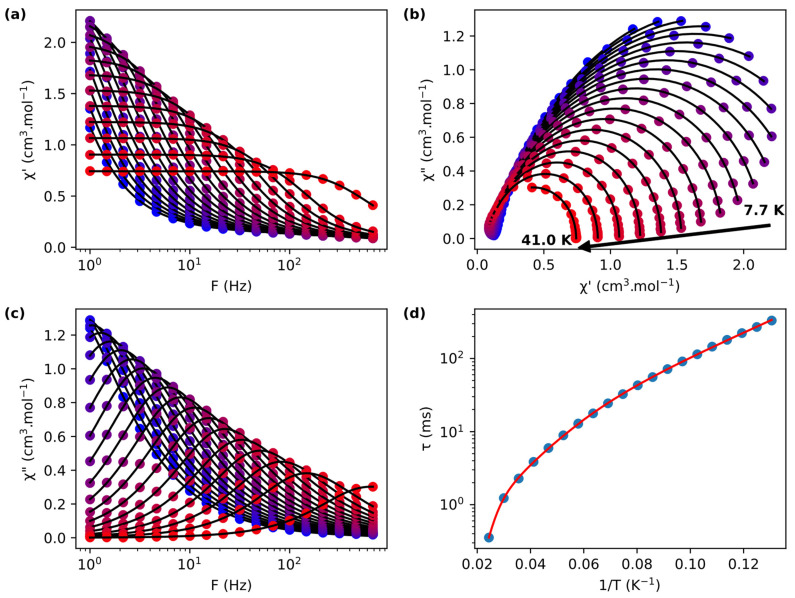
Frequency dependence of the in-phase, χ’, (**a**) and out-of-phase, χ” (**c**) components of the AC susceptibility for **4** in zero DC magnetic field. The red lines are the result of the Cole–Cole fitting. (**b**) Cole–Cole plots obtained using the frequency dependence of χ” for **4**. The solid lines correspond to the best fit obtained with a generalized Debye model. The color gradient, which ranges from blue to red, represents the variation in temperature, with values ranging from 7.7 to 41.0 K, respectively. (**d**) Temperature dependence of the relaxation time for **4** and the corresponding fit with Equation (4) (red solid line).

In contrast to the dynamic magnetic behavior of the mononuclear compounds **1**–**3**, the in-phase, *χ*’, and the out-of-phase, *χ*’’, components of the AC susceptibility for dinuclear complex **4** present a significant frequency-dependent signal in a zero-DC field indicating the presence of the magnetic relaxation (Figure 6a–d). The Cole–Cole plot can be fitted with a generalized Debye model giving *α* parameter of 0.1 coherent with a certain distribution of the relaxation time expected from the frequency dependence of *χ*’’. The dependence of the relaxation time as a function of temperature (Figure 6d) displays a behavior that cannot be fitted by Equation (3). A phenomenological equation comprising three exponential relaxation times has previously been put forth in the literature [35].

(4) $$\tau^{-1}=\tau_{0}^{-1}\exp\left(-\frac{E}{k_{B}T}\right)+\alpha \exp\left(-\frac{\Delta_{1}}{k_{B}T}\right)+\beta \exp\left(-\frac{\Delta_{2}}{k_{B}T}\right)$$

where the first term represents the Orbach relaxation process and the subsequent two terms represent phenomenological terms. The complete set of fitted parameters is presented in Table 3. The *τ*_0_ term is found to be inadequate in comparison to the reported literature, and the high energy barrier *E* of 421 K provides an explanation for the observation of the relaxation time at high temperatures.

As illustrated in Figure 3a, the magnetization curve exhibits a distinctive butterfly-like behavior. A dynamic study of magnetization as a function of the external magnetic field at low temperatures has been conducted, allowing for the extraction of the field-dependent relaxation time. The external magnetic field was swept at a fixed rate of 100 Oe/s, and experiments were conducted at temperatures of 1.8 K, 3.0 K, 4.0 K, and 5.0 K. The curves have been described using a theoretical model, the details of which can be found in the ESI. Figure S6 shows the correlation between the experimental data and the model fitting for the hysteresis loops obtained at different temperatures. The simulation reveals three principal behaviors. Initially, the molecules exhibit pronounced magnetic anisotropy. Secondly, the magnetic easy axis, which is influenced by the presence of strong magnetic anisotropy, demonstrates a tendency to align moderately with the direction of the external magnetic field rather than exhibiting a more randomized distribution. Thirdly, a robust resonant quantum tunneling relaxation time, estimated to be on the order of 1, is identified as the origin of the distinctive butterfly-shaped magnetic curve. All fitted parameters are presented in Table S2 for reference.

### 2.3. Photoluminescence

**Figure 7:**
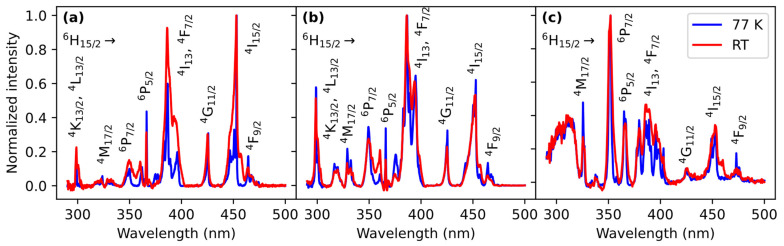
Solid-state excitation spectra for samples **1** (**a**), **2** (**b**), and **3** (**c**) obtained at 77 K and room temperature. The monitoring wavelength was 572 nm for all compounds.

**Figure 8:**
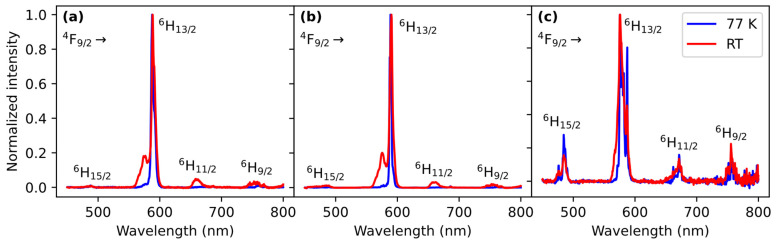
Solid-state emission spectra for samples **1** (**a**), **2** (**b**), and **3** (**c**) obtained at two different temperatures: 77 K and room temperature. The excitation wavelength was 386 nm for all compounds.

The photoluminescence of compounds **1**–**4** was investigated in solid state at 77 K and at room temperature (300 K) (Figure 7 and Figure 8). While dinuclear compound **4** did not show luminescence even at low temperatures, mononuclear complexes **1**–**3** present Dy^3+^ characteristic green–yellow emission under excitation in the UV region. The excitation spectra of **1**–**3** in the 250–500 nm window recorded at 77 K and room temperature monitoring the emission at ca. 572 nm (main ^4^F_9/2_→^6^H_13/2_ transition) shows the characteristic Dy^3+^ *f* − *f* transitions with higher intensities at 450 nm (^6^H_15/2_→^6^I_15/2_), 350 nm (^6^H_15/2_→^6^P_7/2_) and 382 nm (^6^H_15/2_→^6^F_7/2_) for respectively **1**, **2** and **3** (Figure 7). The magnified excitation spectra at 77 K show four component bands of the ^6^H_15/2_ → ^4^F_9/2_ transition at 464, 467, 469, and 474 nm. Such observation indicates that the ligands do not provide an efficient sensitization of Dy^3+^ emission.

The solid-state emission spectra for **1**–**3** performed at 77 K and at room temperature were measured between 450 and 800 nm under excitation at the main excitation bands according to the maximum intensity recorded in the respective excitation spectra (Figure 8). They demonstrate a series of the characteristic Dy^3+^ 4f transitions ^4^F_9/2_→^6^H_9/2_ (between 740 and 770 nm), ^4^F_9/2_→^6^H_11/2_ (between 660 and 680 nm), ^4^F_9/2_→^6^H_13/2_ (between 570 and 590 nm) and ^4^F_9/2_→^6^H_15/2_ (between 470 and 490 nm), corresponding to emissions in the red, yellow–green, and blue spectral windows, respectively. Note that the emission spectra profiles are similar for the 6-coordinated compounds **1** and **3**, with the dominant ^4^F_9/2_→^6^H_13/2_ transition. The emission spectrum for the 5-coordinated **2** is slightly different with a relatively complex profile of the hypersensitive ^4^F_9/2_→^6^H_13/2_ transition, which depends on the local symmetry of Dy^3+^ ion and much more pronounced ^4^F_9/2_→^6^H_15/2_ transition [36]. The occurrence of the luminescence for these mononuclear compounds makes these SMMs multifunctional.

## 3. Materials and Methods

### 3.1. General Procedures

All operations were carried out under an atmosphere of argon using Schlenk techniques or in a nitrogen-filled glovebox. After drying over KOH, THF was purified by distillation from sodium/benzophenone ketyl. Hexane was dried over Na/K alloy, transferred under vacuum, and stored in the glovebox. Pyridine and 2,6-Me_2_C_5_H_3_N were purified by double distillation from CaH_2_. Ph_3_COH was purchased from Acros. Dy(OCPh_3_)_2_(THF)_4_[BPh_4_] [27], Dy(o-Me_2_NC_6_H_4_CH_2_)_3_ [37], DyCl_3_ [38] and [HNEt_3_][BPh_4_] [39] were synthesized according to literature procedure. Lanthanide metal analysis was carried out by complexometric titration [40]. Elemental analysis was performed in the IOMC microanalytical laboratory. IR spectra were recorded as Nujol mulls on a Bruker-Vertex 70 spectrophotometer.

### 3.2. Syntheses

**Synthesis of [Dy(OCPh_3_)_2_(THF)_4_][BPh_4_]·(2,6-Me_2_C_5_H_3_N) (1)**. [Dy(OCPh_3_)_2_(THF)_4_] [BPh_4_] (0.40 g, 0.31 mmol) was dissolved in 5 mL of 2,6-Me_2_C_5_H_3_N. The reaction mixture was stirred for 24 h at ambient temperature. Slow condensation of hexane into the resulting solution at room temperature afforded colorless crystals of **1** in 89% yield (0.39 g). Elemental analysis calcd. (%) for C_85_H_91_BDyNO_6_ (1395.97 g/mol): C 73.13, H 6.57, Dy 11.64, N 1.00; found C 73.45, H 6.81, Dy 11.87, N 1.18. IR (Nujol, KBr) ν/cm^−1^: 1957 (w), 1884 (w), 1812 (w), 1658 (w), 1625 (w), 1592 (s), 1579 (s), 1311 (m), 1264 (m), 1225 (w), 1196 (w), 1175 (m), 1159 (s), 1149 (s), 1093 (s), 1060 (s), 1031 (s), 973 (w), 942 (w), 916 (w), 899 (m), 870 (m), 848 (m), 804 (w), 780 (s), 769 (s), 746 (s), 730 (s), 703 (s), 639 (s), 611 (s), 559 (w), 472 (s).

**Synthesis of *trans*-Dy(OCPh_3_)_3_(THF)_2_ (2).** A solution of Dy(o-Me_2_NC_6_H_4_CH_2_)_3_ (0.42 g, 0.74 mmol) in 20 mL of THF was added to a solution of Ph_3_COH (0.58 g, 2.23 mmol) in 20 mL of THF. The reaction mixture was stirred at ambient temperature overnight. The volatiles were removed in a vacuum. Recrystallization of the solid residue from the THF/hexane (1:8) mixture affords **3** as colorless crystals in 80% yield (0.64 g). Elemental analysis calcd. (%) for C_65_H_61_DyO_5_ (1084.70 g/mol): C 71.98, H 5.67, Dy 14.98; found C 72.17, H 5.89, Dy 15.10. IR (Nujol, KBr) ν/cm^−1^: 1950 (m), 1807 (m), 1595 (s), 1580 (m), 1548 (w), 1487 (s), 1443 (s), 1341 (w), 1311 (s), 1283 (m), 1261 (w), 1246 (w), 1196 (s), 1182 (s), 1159 (s), 1147 (s), 1101 (s), 1064 (s), 1029 (s), 1003 (m), 942 (m), 935 (m), 916 (s), 894 (c), 871 (s), 850 (m), 783 (s), 768 (s), 753 (c), 721 (w), 701 (s), 651 (s), 638 (s), 534 (w), 524 (w), 498 (s), 473 (s).

**Synthesis of *fac*-Dy(OCPh_3_)_3_(py)_3_ (3).** Dy(OCPh_3_)_3_(THF)_2_ (**2**) (0.40 g, 0.37 mmol) was dissolved in 5 mL of pyridine. The reaction mixture was stirred for 1 h at ambient temperature. Slow condensation of hexane into the resulting solution at room temperature afforded colorless crystals of **4** in 79% yield (0.32 g). Elemental analysis calcd. (%) for C_72_H_60_DyN_3_O_3_ (1177.79 g/mol): C 73.42, H 5.14, Dy 13.80, N 3.57; found C 73.17, H 5.39, Dy 13.60, N 3.62. IR (Nujol, KBr) ν/cm^−1^: 1617 (w), 1597 (s), 1569 (w), 1484 (s), 1313 (s), 1260 (s), 1228 (w), 1214 (m), 1204 (m), 1178 (m), 1158 (s), 1115 (m), 1092 (s), 1067 (s), 1036 (s), 1029 (s), 1004 (s), 978 (w), 940 (m), 929 (w), 919 (w), 895 (s), 866 (s), 850 (w), 802 (s), 780 (s), 768 (s), 752 (s), 739 (s), 729 (s), 702 (s), 642 (s), 628 (s), 618 (s), 538 (w), 478 (s).

**Synthesis of [(Ph_3_CO)Dy(THF){(μ^2^-Cl)_2_Li(THF)_2_}μ^2^-Cl]_2_ (4).** To a solution of Ph_3_COH (0.50 g, 1.92 mmol) in 30 mL of diethyl ether n-BuLi (1.6 M hexane solution; 1.2 mL, 1.92 mmol) was added at 0 °C. The reaction mixture was slowly heated up to room temperature and was stirred for 1 h. The solvent was removed in a vacuum. White solid residue was suspended in 35 mL of THF and was added to a suspension of DyCl_3_ (0.52 g, 1.92 mmol) in 5 mL of THF. The reaction mixture was refluxed for 80 h. The solvent was removed in a vacuum, and the solid residue was dissolved in 50 mL of toluene and filtered. Toluene was evaporated in a vacuum, and the solid residue was dissolved in THF (10 mL). Slow condensation of hexane (35 mL) into THF solution at room temperature afforded **4** as colorless crystals in 75% yield (1.08 g). Elemental analysis calcd. (%) for C_62_H_78_Cl_6_Dy_2_Li_2_O_8_ (1502.88 g/mol): C 49.55, H 5.23, Dy 21.63; found C 49.25, H 5.01, Dy 21.90. IR (Nujol, KBr) ν/cm^−1^: 1690 (w), 1875 (w), 1818 (w), 1686 (w), 1595 (m), 1489 (s), 1346 (m), 1320 (m), 1298 (m), 1284 (w), 1261 (w), 1248 (w), 1202 (s), 1179 (s), 1158 (s), 1096 (s), 1065 (s), 1029 (s), 1017 (s), 956 (m), 943 (m), 919 (s), 898 (s), 869 (s), 791 (s), 769 (s), 759 (s), 702 (s), 675 (m), 657 (m), 634 (s), 618 (w), 584 (w), 533 (w), 501 (w), 478 (s).

### 3.3. X-Ray Crystallography

X-ray diffraction data for **1** and **3** were collected at 120 K with a Bruker APEX2 DUO diffractometer, and for **2** and **4**, at 100 K with a Bruker Quest D8 CMOS diffractometer, using graphite monochromated Mo-Kα radiation (λ = 0.71073 Å, ω-scans). Structures were solved using Intrinsic Phasing with the ShelXT [41] structure solution program in Olex2 [42] and then refined with the XL [41] refinement package using Least-Squares minimization against F^2^ in the anisotropic approximation for non-hydrogen atoms. Positions of hydrogen atoms were calculated, and they were refined in the isotropic approximation within the riding model. In **3**, one of the phenyl rings is disordered by two positions with the occupancies refined to 0.58(4) and 0.42(4) using AFIX66 to constrain its geometry; the ADPs of the two components were set equal. In **4**, the occupancies of the two components of the disordered fragment LiCl_2_(THF)_2_ were refined to 0.5089(15) and 0.4911(15) with the ADPs and the bond lengths set to be equal with EADP and SADI, respectively; the occupancies of the carbon atoms of another disordered THF were refined to 0.544(3) and 0.456(3) in a similar manner. Crystal data and structure refinement parameters are given in Table S1. CCDC 2,163,005 (**1**), 2,163,006 (**2**), 2,163,004 (**3**), and 2,387,498 (**4**) contain the supplementary crystallographic data for this paper.

### 3.4. Magnetic Measurements

Magnetic measurements were performed using a Quantum Design MPMS-XL SQUID magnetometer working between 1.8–350 K with a magnetic field of up to 7 Tesla. The samples were prepared in the glovebox. The data were corrected for the sample holder, and the diamagnetic contributions were calculated from Pascal’s constants.

### 3.5. Photoluminescence Spectra

Spectra of optical excitation and luminescence in the visible and NIR regions were recorded using the Fluorolog QM spectrofluorometer, operating in the range of wavelengths from 300 to 1700 nm. Measurements were performed at room temperature (300 K) for crystalline and powdered samples enclosed in sealed quartz capillaries. For low-temperature measurements, powdered samples were cooled to 77 K using a nitrogen-cooled quartz cryostat. A xenon arc lamp, included in the Fluorolog QM setup, served as the continuous excitation source. The samples were prepared in the glovebox.

## 4. Conclusions

In summary, by employing both alkane elimination and salt metathesis approaches and using the strongly coordinating monoanionic Ph_3_CO^−^ ligand in combination with weaker THF or pyridine co-ligands, we synthesized a series of new neutral and cationic mono- and dinuclear complexes. Fine-tuning the reaction conditions led to the formation of complexes featuring Dy^3+^ ions in five- and six-coordinate geometries. Attempts to replace THF with pyridine in the Dy^3+^ coordination sphere of *trans*-Dy(OCPh_3_)_3_(THF)_2_ revealed the structural non-rigidity of the tris(alkoxide) skeleton, which, upon recrystallization from pyridine, undergoes, along with an increase in the coordination number, a change in the mutual arrangement of the alkoxide ligands to form *fac*-Dy(OCPh_3_)_3_(py)_3_. The structural transformation of the cationic complex [Dy(OCPh_3_)_2_(THF)_4_][BPh_4_] featuring *trans*-arrangement of alkoxide ligands into *cis*-[Dy(OCPh_3_)_2_(THF)_4_][BPh_4_]·(2,6-Me_2_C_5_H_3_N) caused by the formation of a solvate with 2,6-dimethylpyridine and reflecting the movability of the octahedral framework in solution is of particular curiosity.

The three investigated mononuclear complexes, cis-[Dy(OCPh_3_)_2_(THF)_4_][BPh_4_]·(2,6-Me_2_C_5_H_3_N), trans-[Dy(OCPh_3_)_3_(THF)_2_], and fac-[Dy(OCPh_3_)_3_(Py)_3_], exhibit slow relaxation of the magnetization under an applied static magnetic field due to the suppression/reduction of the QTM process. As expected, the energy barriers are relatively modest, reflecting the structural features that show a significant deviation of the axial O–Dy–O angles from linearity. Orbach relaxation dominates in the five-coordinate complex, while the relaxation process in the six-coordinate complexes is primarily governed by direct relaxation mechanisms. In contrast, the investigation of the dynamic behavior of the dinuclear complex [(Ph_3_CO)Dy(THF){(μ_2_–Cl)_2_Li(THF)_2_}μ_2_–Cl]_2_ revealed genuine SMM behavior with a relatively high energy barrier of 421 cm^−1^, although it displayed a modest coercive field of 3.2 kOe. Except for the dinuclear complex, in which the presence of two Dy^3+^ ions appears to quench luminescence, the mononuclear complexes exhibit characteristic Dy^3+^ emissions, underscoring their multifunctional nature. Therefore, the use of strongly coordinated Ph_3_CO^−^ in combination with other weakly coordinated ligands, such as THF or pyridine, was conducted on three field-induced and one dinuclear genuine SMM. However, their SMM’s performance does not exceed one of our previously reported 6-coordinated *trans*-[Dy(OCPh_3_)_2_(THF)_4_][BPh_4_] complex, which highlights the important challenge in the synthetic approach [27].

## Figures and Tables

**Table 1:** Selected bond distances and angles for **1**–**4**.

Compound	Dy−O^−^ Distances (Å)	Dy−O/N Distances (Å)	^−^O−Dy−O^−^ Angle (°)	O_ax_−Dy−X_ax_ Angle (°)
**1**	2.054(4), 2.062(5)	2.341(5)–2.449(6)	102.7(2)	164.4(2)
**2**	1.980(17)–2.102(5)	2.362(5), 2.407(5)	97.8(8)–163.0(10) ^a^	169.55(17)
**3**	2.124(8)	2.601(11)	102.4(3)	161.6(4)
**4**	2.0335(12)	2.3473(15)	-	171.46(7), 164.59(8) ^b^

^a^ Two of the ^−^OCPh_3_ ligands are disordered by two positions. ^b^ Li(THF)_2_Cl_2_ fragment is disordered by two positions.

**Table 2:** Main parameters for dynamic magnetic behavior of compounds **1**–**3**.

Samples	A_T_ (s^−1^.Oe^−4^)	B_1_ (s^−1^)	B_2_ (Oe^−2^)	C_T_ (s^−1^)	A_H_ (s^−1^.K^−1^)	τ_0_ (s^−1^)	E (cm^−1^)	C (s^−1^.K^−9^)
		B (s^−1^.Oe^2^)						
**1**	(2.1 ± 0.2) ×10^−11^	(1.5 ± 1.0) ×10^3^	(3.7 ± 2.5) ×10^−4^	4.2 ± 2.0	22 ± 1	(1.3 ± 0.3) ×10^−5^	11.1 ± 0.4	(1.03 ± 0.07) ×10^−2^
**2**	(1.8 ± 0.4) ×10^−11^	82 ± 57	(2.1 ± 1.8) ×10^−4^	4.2 ± 0.9	2.0 ± 0.1	(2.2 ± 0.5) ×10^−6^	16.5 ± 0.3	(1.27 ± 0.07) ×10^−2^
**3**	(7.1 ± 0.8) ×10^−8^	(5.8 ± 5.4) × 10^5^		81 ± 45	485 ± 4	(2.1 ± 0.8) ×10^−5^	9.4 ± 0.7	(3.7 ± 0.2) ×10^−2^

**Table 3:** Fit parameters for the temperature dependence of the relaxation time performed with Equation (4) for compound **4**.

$\tau_{0}$ (s^−1^)	E (K)	α (s^−1^)	$\Delta_{1}$ (K)	β (s^−1^)	$\Delta_{2}$ (K)
(2.0 ± 1.2) 10^−8^	421 ± 25	(1.0 ± 0.1) 10^−4^	98 ± 3	(2.6 ± 0.2) 10^−3^	37.5 ± 0.7

## Data Availability

The original contributions presented in the study are included in the article/supplementary material; further inquiries can be directed to the corresponding authors.

## Supplementary Materials

The following supporting information can be downloaded at: https://www.mdpi.com/article/10.3390/molecules29225343/s1, Figure S1. Fragments of the crystal packing in 1 (top left), 2 (top right), 3 (bottom left) along the crystallographic axis *c* and in 4 (bottom right) along the crystallographic axis *a*. Hydrogen atoms and minor components of the disordered ligands have been omitted for clarity; Figure S2. Frequency dependence of χ’ (a) and χ” (c) for 2 at 1.8 K performed under various applied dc fields. (b) Cole-Cole plots obtained using the frequency dependence of χ” for 2 at 1.8 K under various dc field. The solid lines correspond to the best fit obtained with a generalized Debye model. (d) Field dependence of the relaxation time for 2. The red line represents the fit using Equation (1); Figure S3. Frequency dependence of the in-phase, χ’, (a) and out-of-phase, χ” (c) components of the ac susceptibility for 2 under optimal applied magnetic field of 500 Oe. The black lines are the result of the Cole-Cole fitting. (b) Cole-Cole plots obtained using the frequency dependence of χ” for 2 obtained under 500 Oe. The solid lines correspond to the best fit obtained with a generalized Debye model. (d) Temperature dependence of the relaxation time for 2 (500 Oe) and the corresponding fit with Equation (3) (red solid line); Figure S4. Frequency dependence of χ’ (a) and χ” (c) for 3 at 1.8 K performed under various applied dc fields. (b) Cole-Cole plots obtained using the frequency dependence of χ” for 3 at 1.8 K under various dc field. The solid lines correspond to the best fit obtained with a generalized Debye model. (d) Field dependence of the relaxation time for 3. The red line represents the fit using Equation (1); Figure S5. Frequency dependence of the in-phase, χ’, (a) and out-of-phase, χ” (c) components of the ac susceptibility for 3 under optimal applied magnetic field of 200 Oe. The black lines are the result of the Cole-Cole fitting. (b) Cole-Cole plots obtained using the frequency dependence of χ” for 3 obtained under 200 Oe. The solid lines correspond to the best fit obtained with a generalized Debye model. (d) Temperature dependence of the relaxation time for 3 (200 Oe) and the corresponding fit with Equation (3) (red solid line); Figure S6. Magnetization as a function of the external magnetic field for sample 4, with a range between -2 Tesla and 2 Tesla and a variation field speed of 100 Oe.s^−1^ at (a) 1.8 K, (b) 3 K, (c) 4 K and (d) 5 K. The plein circles represent the experimental points, and the orange lines represent the theoretical fit; Figure S7. Density of probability of the angle between the magnetic easy axis of the molecules and the external magnetic field as a function of this angle with *σ* = 1.327 rad; Table S1. Crystal data, data collection and structure refinement details for 1–4; Table S2. Fit parameters for the field dependence of the magnetization performed with Equations (5) and (12) for compound **4**.
