# Supplementary material for: Multifunctional Dy3+ Complexes with Triphenylmethanolates: Structural Diversity, Luminescence, and Magnetic Relaxation
Source: Molecules. 2024 Nov 13;29(22):5343. doi: 10.3390/molecules29225343 (PMC11596367; doi:10.3390/molecules29225343)

checkCIF/PLATON report

Structure factors have been supplied for datablock(s) 1

THIS REPORT IS FOR GUIDANCE ONLY. IF USED AS PART OF A REVIEW PROCEDURE FOR PUBLICATION, IT SHOULD NOT REPLACE THE EXPERTISE OF AN EXPERIENCED CRYSTALLOGRAPHIC REFEREE.

No syntax errors found.   CIF dictionary   Interpreting this report

Datablock: 1

Bond precision: C-C = 0.0140 A   Wavelength=0.71073

Cell:	a=24.162(10)	b=12.664(5)	c=25.40(1)
	alpha=90	beta=114.173(9)	gamma=90
Temperature:	120 K		

	Calculated	Reported
Volume	7091(5)	7090(5)
Space group	P 21/n	P 1 21/n 1
Hall group	-P 2yn	-P 2yn
Moiety formula	C54 H62 Dy O6, C24 H20 B, C7 H9 N	C54 H62 Dy O6, C7 H9 N, C24 H20 B
Sum formula	C85 H91 B Dy N O6	C85 H91 B Dy N O6
Mr	1395.90	1395.89
Dx,g cm-3	1.308	1.308
Z	4	4
Mu (mm-1)	1.109	1.109
F000	2908.0	2908.0
F000'	2908.25	
h,k,lmax	29,15,31	29,15,31
Nref	13945	13927
Tmin,Tmax	0.936,0.946	0.060,0.100
Tmin'	0.758	

Correction method= # Reported T Limits: Tmin=0.060 Tmax=0.100
AbsCorr = MULTI-SCAN

Data completeness= 0.999   Theta(max)= 25.999

R(reflections)= 0.0660(6880)   wR2(reflections)= 0.1625(13927)

S = 0.933   Npar= 850

The following ALERTS were generated. Each ALERT has the format
test-name_ALERT_alert-type_alert-level.
Click on the hyperlinks for more details of the test.

Alert level B

```
RINTA01_ALERT_3_B  The value of Rint is greater than 0.18
            Rint given   0.217
```

Author Response: The best available crystal was a thin needle of a low reflective power

Alert level C

```
PLAT026_ALERT_3_C Ratio Observed / Unique Reflections (too) Low ..        49% Check
PLAT042_ALERT_1_C Calc. and Reported MoietyFormula Strings  Differ     Please Check
              Calc: C54 H62 Dy O6, C24 H20 B, C7 H9 N
              Rep.: C54 H62 Dy O6, C7 H9 N, C24 H20 B
PLAT230_ALERT_2_C Hirshfeld Test Diff for    C18      --C19      .        5.1 s.u.
PLAT234_ALERT_4_C Large Hirshfeld Difference N1       --C64      .       0.19 Ang.
PLAT234_ALERT_4_C Large Hirshfeld Difference C65      --C66      .       0.20 Ang.
PLAT241_ALERT_2_C High   'MainMol' Ueq as Compared to Neighbors of       C15S Check
PLAT244_ALERT_4_C Low    'Solvent' Ueq as Compared to Neighbors of        C68 Check
PLAT342_ALERT_3_C Low Bond Precision on  C-C Bonds ..............    0.01395 Ang.
PLAT911_ALERT_3_C Missing FCF Refl Between Thmin & STh/L=    0.600         15 Report
                5  1  1,   3 14  2,  24  1  2,  -1  1  6,   5 12 11,  18  0 12,
              -22  8 15, -26  2 19,  10  0 20, -15  8 23, -20  4 25, -19  5 25,
              -12  7 25, -11  7 25, -10  7 25,
PLAT975_ALERT_2_C Check Calcd Resid. Dens.  1.10Ang From O5      .       0.52 eA-3
PLAT976_ALERT_2_C Check Calcd Resid. Dens.  1.09Ang From O5      .      -0.42 eA-3
```

Alert level G

```
PLAT020_ALERT_3_G The Value of Rint is Greater Than 0.12 ........      0.217 Report
PLAT720_ALERT_4_G Number of Unusual/Non-Standard Labels ..........        18 Note
              H1SA    H1SB    H2SA    H2SB    H3SA    H3SB    H4SA    H4SB
              H5SA    H5SB    H6SA    H6SB    H7SA    H7SB    H8SA    H8SB
              H9SA    H9SB
PLAT794_ALERT_5_G Tentative Bond Valency for Dy1        (III)     .       3.11 Info
PLAT883_ALERT_1_G No Info/Value for _atom_sites_solution_primary .     Please Do !
PLAT910_ALERT_3_G Missing # of FCF Reflection(s) Below Theta(Min).         3 Note
               -1  0  1,   1  0  1,   0  0  2,
PLAT933_ALERT_2_G Number of HKL-OMIT Records in Embedded .res File         2 Note
               -1  0  1,   1  0  1,
PLAT941_ALERT_3_G Average HKL Measurement Multiplicity ..........        4.9 Low
PLAT960_ALERT_3_G Number of Intensities with I  <   - 2*sig(I) ...         7 Check
PLAT967_ALERT_5_G Note: Two-Theta Cutoff Value in Embedded .res ..      52.0 Degree
PLAT969_ALERT_5_G The 'Henn et al.' R-Factor-gap value ..........      1.080 Note
              Predicted wR2: Based on SigI**2 15.05 or SHELX Weight 17.41
PLAT978_ALERT_2_G Number C-C Bonds with Positive Residual Density.         0 Info
```

0 **ALERT level A** = Most likely a serious problem - resolve or explain
1 **ALERT level B** = A potentially serious problem, consider carefully

```
11 ALERT level C = Check. Ensure it is not caused by an omission or oversight
11 ALERT level G = General information/check it is not something unexpected

 2 ALERT type 1 CIF construction/syntax error, inconsistent or missing data
 6 ALERT type 2 Indicator that the structure model may be wrong or deficient
 8 ALERT type 3 Indicator that the structure quality may be low
 4 ALERT type 4 Improvement, methodology, query or suggestion
 3 ALERT type 5 Informative message, check
```

It is advisable to attempt to resolve as many as possible of the alerts in all categories. Often the minor alerts point to easily fixed oversights, errors and omissions in your CIF or refinement strategy, so attention to these fine details can be worthwhile. In order to resolve some of the more serious problems it may be necessary to carry out additional measurements or structure refinements. However, the purpose of your study may justify the reported deviations and the more serious of these should normally be commented upon in the discussion or experimental section of a paper or in the "special_details" fields of the CIF. checkCIF was carefully designed to identify outliers and unusual parameters, but every test has its limitations and alerts that are not important in a particular case may appear. Conversely, the absence of alerts does not guarantee there are no aspects of the results needing attention. It is up to the individual to critically assess their own results and, if necessary, seek expert advice.

Publication of your CIF in IUCr journals

A basic structural check has been run on your CIF. These basic checks will be run on all CIFs submitted for publication in IUCr journals (*Acta Crystallographica*, *Journal of Applied Crystallography*, *Journal of Synchrotron Radiation*); however, if you intend to submit to *Acta Crystallographica Section C* or *E* or *IUCrData*, you should make sure that full publication checks are run on the final version of your CIF prior to submission.

Publication of your CIF in other journals

Please refer to the *Notes for Authors* of the relevant journal for any special instructions relating to CIF submission.

PLATON version of 13/05/2024; check.def file version of 04/05/2024

Datablock 1 - ellipsoid plot

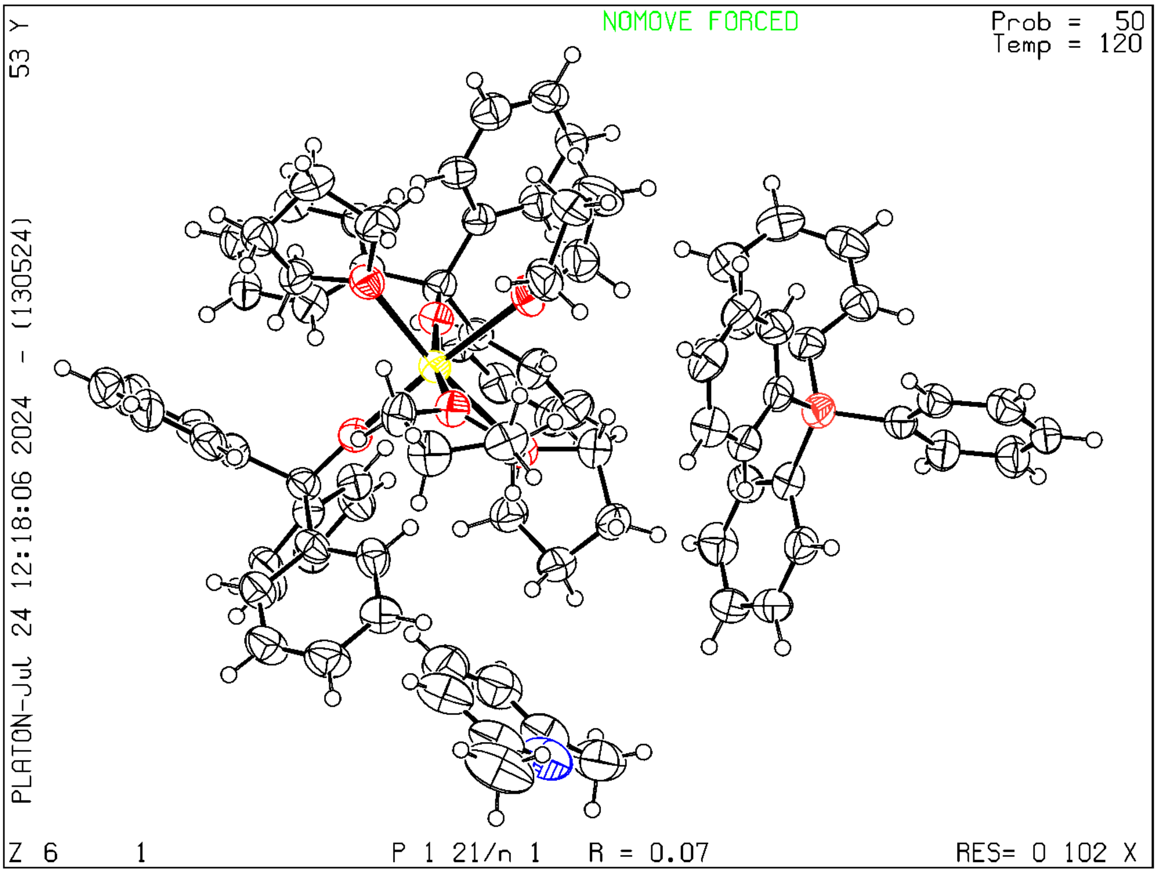

Supplement: Supplementary file 1 [file molecules-29-05343-s001.zip › checkcif_1.pdf]
