# Supplementary material for: Multifunctional Dy3+ Complexes with Triphenylmethanolates: Structural Diversity, Luminescence, and Magnetic Relaxation
Source: Molecules. 2024 Nov 13;29(22):5343. doi: 10.3390/molecules29225343 (PMC11596367; doi:10.3390/molecules29225343)

checkCIF/PLATON report

Structure factors have been supplied for datablock(s) 4

THIS REPORT IS FOR GUIDANCE ONLY. IF USED AS PART OF A REVIEW PROCEDURE FOR PUBLICATION, IT SHOULD NOT REPLACE THE EXPERTISE OF AN EXPERIENCED CRYSTALLOGRAPHIC REFEREE.

No syntax errors found. CIF dictionary Interpreting this report

Datablock: 4

```
Bond precision:   C-C = 0.0030 A          Wavelength=0.71073

Cell:           a=10.0466(2)        b=10.8954(2)      c=16.9455(3)
                alpha=103.346(1)    beta=99.693(1)    gamma=109.892(1)
Temperature:    100 K

                 Calculated                  Reported
Volume           1634.14(6)                  1634.14(5)
Space group      P -1                        P -1
Hall group       -P 1                        -P 1
Moiety formula   C62 H78 Cl6 Dy2 Li2 O8      C62 H78 Cl6 Dy2 Li2 O8
Sum formula      C62 H78 Cl6 Dy2 Li2 O8      C62 H78 Cl6 Dy2 Li2 O8
Mr               1502.83                     1502.82
Dx,g cm-3        1.527                       1.527
Z                1                           1
Mu (mm-1)        2.564                       2.564
F000             754.0                       754.0
F000'            754.75
h,k,lmax         12,13,21                    12,13,21
Nref             7132                        7121
Tmin,Tmax        0.743,0.774                 0.260,0.347
Tmin'            0.593

Correction method= # Reported T Limits: Tmin=0.260 Tmax=0.347
AbsCorr = MULTI-SCAN

Data completeness= 0.998           Theta(max)= 26.998

R(reflections)= 0.0176( 6930)                   wR2(reflections)=
                                                0.0416( 7121)
S = 1.044                    Npar= 412
```

The following ALERTS were generated. Each ALERT has the format
test-name_ALERT_alert-type_alert-level.
Click on the hyperlinks for more details of the test.

Alert level C

```
PLAT220_ALERT_2_C NonSolvent   Resd 1  C   Ueq(max)/Ueq(min) Range        3.1 Ratio
PLAT911_ALERT_3_C Missing FCF Refl Between Thmin & STh/L=    0.600         10 Report
                2  7  5,  -7  8  5,   3  7  6,  -5  9  6,   0  8  7,   3  5  9,
               -6  6  9,  -4  6 10,  -4 -7 12,  -3  4 12,
```

Alert level G

```
PLAT002_ALERT_2_G Number of Distance or Angle Restraints on AtSite         30 Note
PLAT003_ALERT_2_G Number of Uiso or U(i,j) Restrained non-H Atoms          11 Report
PLAT154_ALERT_1_G The s.u.'s on the Cell Angles are Equal ..(Note)      0.001 Degree
PLAT171_ALERT_4_G The CIF-Embedded .res File Contains EADP Records         17 Report
PLAT176_ALERT_4_G The CIF-Embedded .res File Contains SADI Records         20 Report
PLAT186_ALERT_4_G The CIF-Embedded .res File Contains ISOR Records          5 Report
PLAT230_ALERT_2_G Hirshfeld Test Diff for    O2        --C20      .      14.9 s.u.
PLAT230_ALERT_2_G Hirshfeld Test Diff for    O2        --C20'     .      17.7 s.u.
PLAT232_ALERT_2_G Hirshfeld Test Diff (M-X)  Dy1       --Cl1      .       5.5 s.u.
PLAT232_ALERT_2_G Hirshfeld Test Diff (M-X)  Dy1       --Cl1_a    .       7.5 s.u.
PLAT301_ALERT_3_G Main Residue  Disorder ..............(Resd    1)        40% Note
PLAT811_ALERT_5_G No ADDSYM Analysis: Too Many Excluded Atoms ....          ! Info
PLAT860_ALERT_3_G Number of Least-Squares Restraints ............          83 Note
PLAT910_ALERT_3_G Missing # of FCF Reflection(s) Below Theta(Min).          1 Note
                0  0  1,
PLAT941_ALERT_3_G Average HKL Measurement Multiplicity ..........         2.6 Low
PLAT967_ALERT_5_G Note: Two-Theta Cutoff Value in Embedded .res ..       54.0 Degree
PLAT969_ALERT_5_G The 'Henn et al.' R-Factor-gap value ..........       1.511 Note
              Predicted wR2: Based on SigI**2  2.73 or SHELX Weight  3.98
PLAT978_ALERT_2_G Number C-C Bonds with Positive Residual Density.         13 Info
```

0 **ALERT level A** = Most likely a serious problem - resolve or explain
0 **ALERT level B** = A potentially serious problem, consider carefully
2 **ALERT level C** = Check. Ensure it is not caused by an omission or oversight
18 **ALERT level G** = General information/check it is not something unexpected

1 ALERT type 1 CIF construction/syntax error, inconsistent or missing data
8 ALERT type 2 Indicator that the structure model may be wrong or deficient
5 ALERT type 3 Indicator that the structure quality may be low
3 ALERT type 4 Improvement, methodology, query or suggestion
3 ALERT type 5 Informative message, check

It is advisable to attempt to resolve as many as possible of the alerts in all categories. Often the minor alerts point to easily fixed oversights, errors and omissions in your CIF or refinement strategy, so attention to these fine details can be worthwhile. In order to resolve some of the more serious problems it may be necessary to carry out additional measurements or structure refinements. However, the purpose of your study may justify the reported deviations and the more serious of these should normally be commented upon in the discussion or experimental section of a paper or in the "special_details" fields of the CIF. checkCIF was carefully designed to identify outliers and unusual parameters, but every test has its limitations and alerts that are not important in a particular case may appear. Conversely, the absence of alerts does not guarantee there are no aspects of the results needing attention. It is up to the individual to critically assess their own results and, if necessary, seek expert advice.

Publication of your CIF in IUCr journals

A basic structural check has been run on your CIF. These basic checks will be run on all CIFs submitted for publication in IUCr journals (*Acta Crystallographica*, *Journal of Applied Crystallography*, *Journal of Synchrotron Radiation*); however, if you intend to submit to *Acta Crystallographica Section C* or *E* or *IUCrData*, you should make sure that full publication checks are run on the final version of your CIF prior to submission.

Publication of your CIF in other journals

Please refer to the *Notes for Authors* of the relevant journal for any special instructions relating to CIF submission.

PLATON version of 22/08/2024; check.def file version of 21/08/2024

Datablock 4 - ellipsoid plot

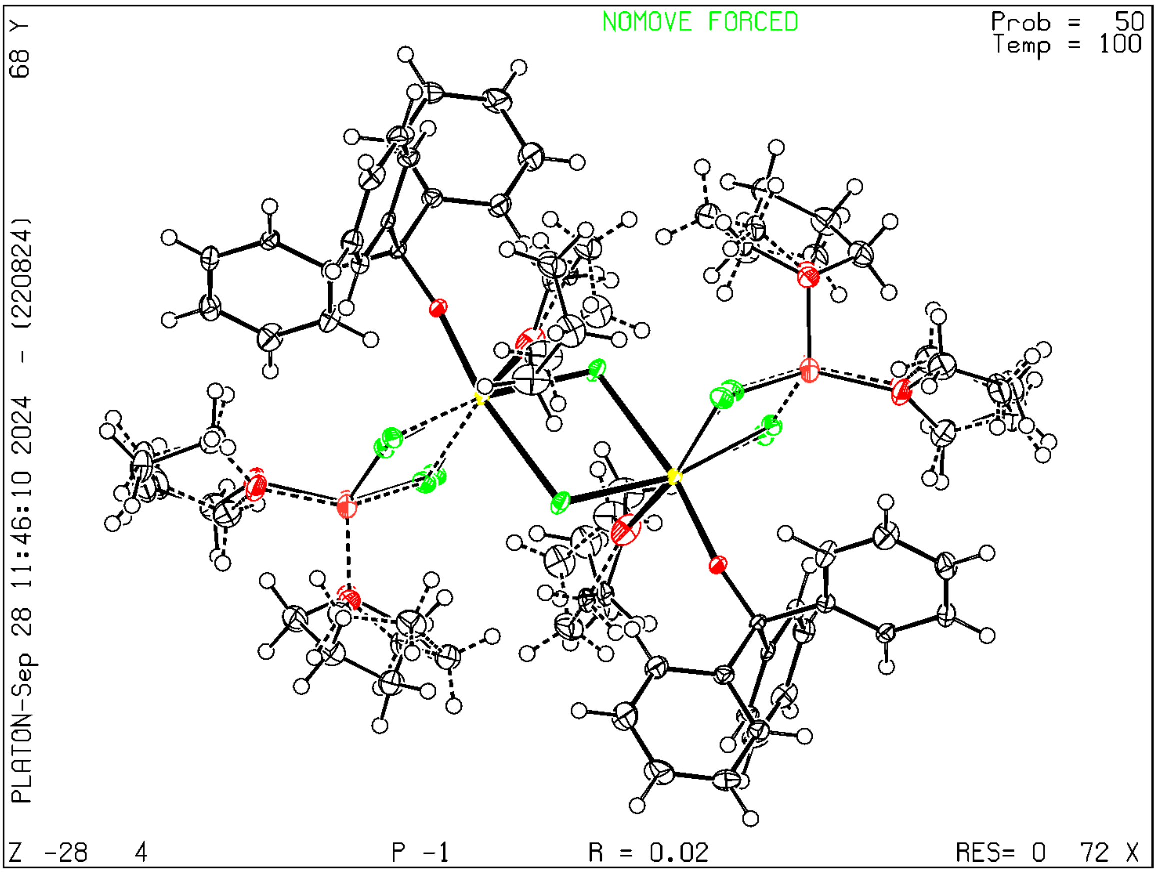

Supplement: Supplementary file 1 [file molecules-29-05343-s001.zip › checkcif_4.pdf]
