# Supplementary material for: Multifunctional Dy3+ Complexes with Triphenylmethanolates: Structural Diversity, Luminescence, and Magnetic Relaxation
Source: Molecules. 2024 Nov 13;29(22):5343. doi: 10.3390/molecules29225343 (PMC11596367; doi:10.3390/molecules29225343)
Supplement: Supplementary file 1 [file molecules-29-05343-s001.zip › ESI Molecules.pdf]

SUPPORTING INFORMATION

Multifunctional Dy^{3+} Complexes with Triphenylalkoxides: Structural Diversity, Luminescence, and Magnetic Relaxation

Gautier Félix,*[1] Aleksei O. Tolpygin,[2] Aurore Larquey,[1] Ilia A. Gogolev,[3] Yulia V. Nelyubina,[3] Yannick Guari,[1] Joulia Larionova*[1] and Alexander A. Trifonov*[2,3]

[1] ICGM, Univ. Montpellier, CNRS, ENSCM, Montpellier, France. E-mail: gautier.felix@umontpellier.fr, joulia.larionova@umontpellier.

[2] G.A. Razuvaev Institute of Organometallic Chemistry of Russian Academy of Sciences, 49 Tropinina str., GSP-445, 603950, Nizhny Novgorod, Russia. E-mail: trif@iomc.ras.ru

[3] A.N. Nesmeyanov Institute of Organoelement Compounds of Russian Academy of Sciences, 28 Vavilova str., bld. 1, 119334, Moscow, Russia.

* Correspondence: G.F.: gautier.felix@umontpellier.fr; J.L.: joulia.larionova@umontpellier.fr; A.A.T.: trif@iomc.ras.ru.

CONTENTS

Model....3

Figures....5

Figure S1. Fragments of the crystal packing in 1 (top left), 2 (top right), 3 (bottom left) along the crystallographic axis *c* and in 4 (bottom right) along the crystallographic axis *a*. Hydrogen atoms and minor components of the disordered ligands have been omitted for clarity....5

Figure S2. Frequency dependence of χ' (a) and χ" (c) for 2 at 1.8 K performed under various applied dc fields. (b) Cole-Cole plots obtained using the frequency dependence of χ" for 2 at 1.8 K under various dc field. The solid lines correspond to the best fit obtained with a generalized Debye model. (d) Field dependence of the relaxation time for 2. The red line represents the fit using Eq. (1)....6

Figure S3. Frequency dependence of the in-phase, χ', (a) and out-of-phase, χ" (c) components of the ac susceptibility for 2 under optimal applied magnetic field of 500 Oe. The black lines are the result of the Cole-Cole fitting. (b) Cole-Cole plots obtained using the frequency dependence of χ" for 2 obtained under 500 Oe. The solid lines correspond to the best fit obtained with a generalized Debye model. (d) Temperature dependence of the relaxation time for 2 (500 Oe) and the corresponding fit with Eq. (3) (red solid line)....7

Figure S4. Frequency dependence of χ' (a) and χ" (c) for 3 at 1.8 K performed under various applied dc fields. (b) Cole-Cole plots obtained using the frequency dependence of χ" for 3 at 1.8 K under various dc field. The solid lines correspond to the best fit obtained with a generalized Debye model. (d) Field dependence of the relaxation time for 3. The red line represents the fit using Eq. (1)....8

Figure S5. Frequency dependence of the in-phase, χ', (a) and out-of-phase, χ" (c) components of the ac susceptibility for 3 under optimal applied magnetic field of 200 Oe. The black lines are the result of the Cole-Cole fitting. (b) Cole-Cole plots obtained using the frequency dependence of χ" for 3 obtained under 200 Oe. The solid lines correspond to the best fit

obtained with a generalized Debye model. (d) Temperature dependence of the relaxation time for 3 (200 Oe) and the corresponding fit with Eq. (3) (red solid line)....9

Figure S6. Magnetization as a function of the external magnetic field for sample 4, with a range between -2 Tesla and 2 Tesla and a variation field speed of 100 Oe.s^{-1} at (a) 1.8 K, (b) 3 K, (c) 4 K and (d) 5 K. The plein circles represent the experimental points, and the orange lines represent the theoretical fit....10

Figure S7. Density of probability of the angle between the magnetic easy axis of the molecules and the external magnetic field as a function of this angle with σ = 1.327 rad....10

Tables....11

Table S1. Crystal data, data collection and structure refinement details for 1 - 4....11

Table S2. Fit parameters for the field dependence of the magnetization performed with Eq. (5) and (12) for compound 4....12

MODEL

The following equation provides an explanation of the magnetization dynamic modelization.

$$\frac{\mathrm{d}M(t)}{\mathrm{d}t} = \frac{\left(M_0(H,T) - M(t)\right)}{\tau(H,T)} \tag{S1}$$

where:

$$\tau^{-1} = \tau_{DIRECT}^{-1} + \tau_{Temp}^{-1} + \sum_{\mathrm{n}=0}^{\mathrm{N}} \tau_{nQTM}^{-1} \tag{S2}$$

τ_{DIRECT} represents the relaxation time for the direct process, which is inversely proportional to the temperature and the external magnetic field to the power of 4 for Kramers ions.

$$\tau_{DIRECT}^{-1} = A_{DIRECT} \cdot H^4 \cdot T \tag{S3}$$

τ_{Temp} is independent of the external magnetic field and encompasses all temperature-related processes, including Raman and Orbach processes.

$$\tau_{Temp}^{-1} = C_{Temp}(T) \tag{S4}$$

τ_{nQTM} represents the resonant quantum tunneling relaxation time at the order n (it is interesting to note that the order 0 is the classical quantum tunneling relaxation).

$$\tau_{nQTM}^{-1} = \frac{B_{n,1}}{1 + B_{n,2} \cdot \left(H \pm n \cdot H_{QTM}\right)^2} \tag{S5}$$

with $B_{n,1}$ and $B_{n,2}$ two constants, and H_{QTM} the resonant magnetic field value.

The stable value of the magnetization at a given field and temperature is represented by $M_0(H,T)$. The experimental value of the magnetic saturation, which is just above 10 $N\beta$, provides two pieces of information. Firstly, spins of both Dy^{3+} ions are aligned along the same axis due to a strong magnetic anisotropy. Secondly, the orientation of the magnetic easy axis is pseudo-randomly oriented along the external magnetic field. This phenomenon can be attributed to the initial random distribution of the easy axes. However, upon the application of an external magnetic field, the molecular clusters exhibit insufficient fixation, leading to a tendency to align mechanically with the external magnetic field. The initial observation, coupled with the fact that only the ±15/2 spin states are populated at low temperatures, gives rise to a reduction in the complexity of the Hamiltonian. In this context, the spins of both Dy^{3+} are not regarded as 15/2, but are instead replaced by a pseudo spin of 1/2 with a pseudo g factor (gx = 0, gy = 0, and gz = 20). The Hamiltonian is written as follows:

$$\mathcal{H} = -J \cdot \widehat{S_{1,z}} \cdot \widehat{S_{2,z}} - g_z \cdot B_0 \cdot \cos(\theta) \cdot \left(\widehat{S_{1,z}} + \widehat{S_{2,z}}\right) \tag{S6}$$

Where $\widehat{S_{1,z}}$ and $\widehat{S_{2,z}}$ represent the pseudo-spin "z" matrices of the first and second dysprosium atoms, respectively, within the molecule. J denotes the magnetic interaction between the two spins, B_0 represents the amplitude of the external magnetic field, and θ denotes the angle between the external magnetic field and the magnetic easy axis. The partition function for the system, as defined by **Hamiltonian (S6)**, is expressed as follows:

$$\mathcal{Z} = \mathrm{Tr}\left(\exp\left(-\frac{\mathcal{H}}{k_B T}\right)\right) \tag{S7}$$

And the average magnetization is equal to:

$$m(\theta) = \mathrm{Tr}\left(g_z \cdot \cos(\theta) \cdot \left(\widehat{S_{1,z}} + \widehat{S_{2,z}}\right) \cdot \exp\left(-\frac{\mathcal{H}}{k_B T}\right)\right) / \mathcal{Z} \tag{S8}$$

And as a function of θ:

$$m(\theta) = g_z \cdot \cos(\theta) \frac{\sinh(g_z \cdot B_0 \cdot \cos(\theta) / (k_B T))}{\cosh(g_z \cdot B_0 \cdot \cos(\theta) / (k_B T)) + \exp(-J/(2 k_B T))} \tag{S9}$$

In the case of an infinite number of molecules where the magnetic easy axis is randomly oriented in space, the total average magnetization can be expressed as follows:

$$M_0(H,T) = \frac{1}{2} \int_{\theta=0}^{\pi} g_z \cdot \cos(\theta) \frac{\sinh(g_z \cdot B_0 \cdot \cos(\theta) / (k_B T))}{\cosh(g_z \cdot B_0 \cdot \cos(\theta) / (k_B T)) + \exp(-J/(2 k_B T))} \sin(\theta)\, \mathrm{d}\theta \tag{S10}$$

However, as we have said previously the magnetic easy axis is pseudo-randomly oriented, which means that the total average magnetization is pondered by a factor $P(\theta)$ expressed as follows:

$$\begin{aligned} M_0(H,T) = \int_{\theta=0}^{\pi} & g_z \cdot \cos(\theta) \\ & \cdot \frac{\sinh(g_z \cdot B_0 \cdot \cos(\theta) / (k_B T))}{\cosh(g_z \cdot B_0 \cdot \cos(\theta) / (k_B T)) + \exp(-J/(2 k_B T))} P(\theta) \sin(\theta)\, \mathrm{d}\theta \end{aligned} \tag{S11}$$

In this context, $P(\theta)$ represents the probability density associated with the angle between the external magnetic field and the magnetic easy axis. For the purposes of this analysis, we will assume that:

$$P(\theta) = \frac{1}{\int_{\theta=0}^{\pi} \left[\exp\left(-\frac{\theta^2}{\sigma^2}\right) + \exp\left(-\frac{(\theta-\pi)^2}{\sigma^2}\right)\right] \sin(\theta)\, \mathrm{d}\theta} \cdot \left[\exp\left(-\frac{\theta^2}{\sigma^2}\right) + \exp\left(-\frac{(\theta-\pi)^2}{\sigma^2}\right)\right] \tag{S12}$$

The parameter σ, which represents the half-width Gaussian, quantifies the degree of alignment between the magnetic easy axis and the external magnetic field. If σ tends to 0, all magnetic easy axes of all molecules are aligned with the external magnetic field. Conversely, if σ approaches infinity, all magnetic easy axes of all molecules are randomly distributed in space.

FIGURES

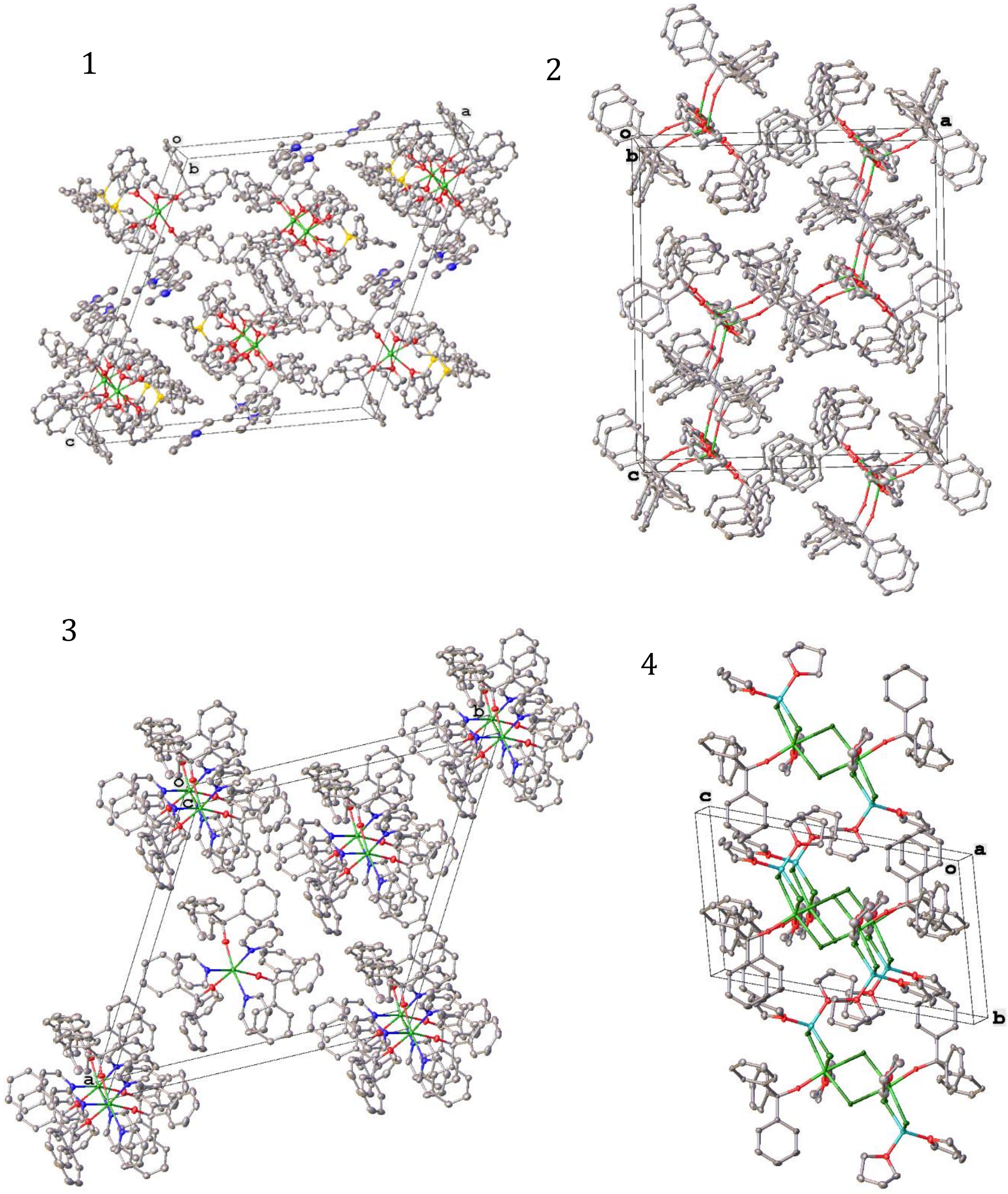

Figure S1. Fragments of the crystal packing in **1** (top left), **2** (top right), **3** (bottom left) along the crystallographic axis *c* and in **4** (bottom right) along the crystallographic axis *a*. Hydrogen atoms and minor components of the disordered ligands have been omitted for clarity.

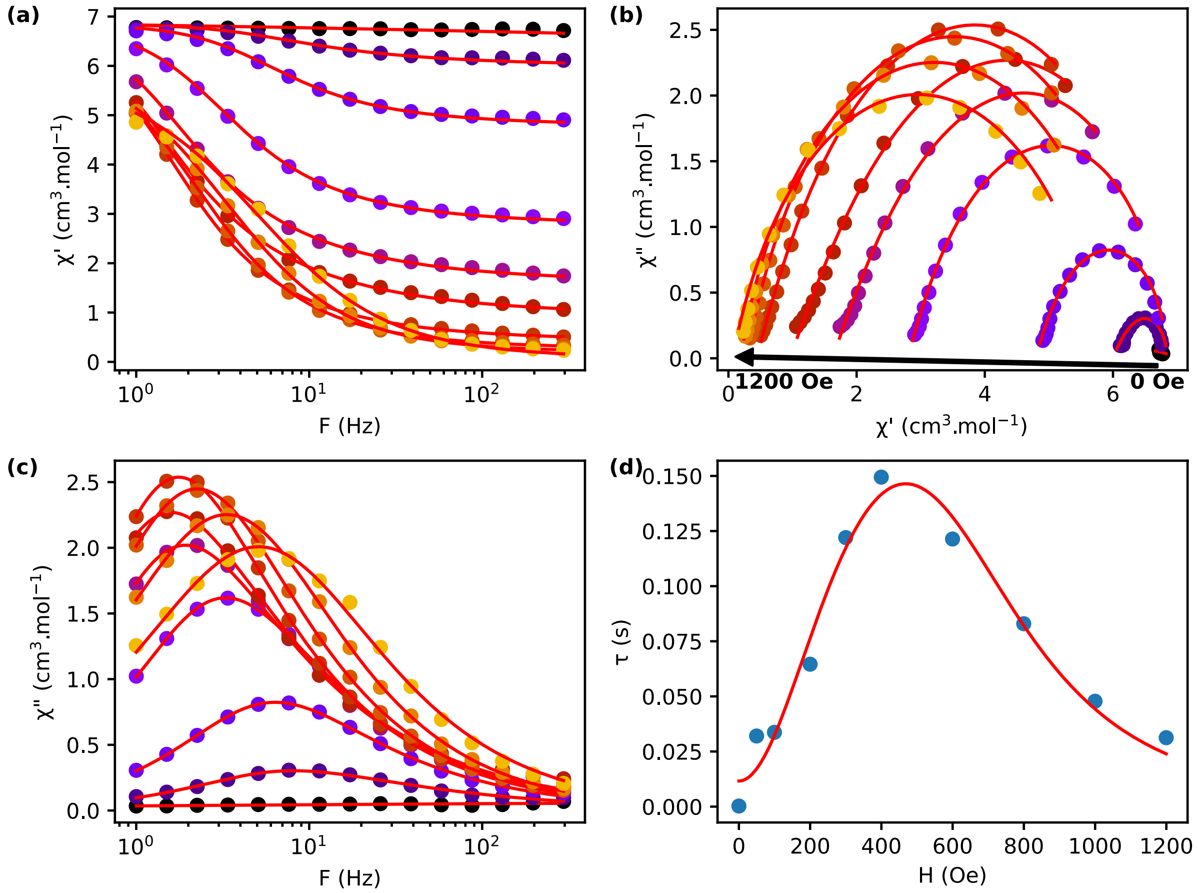

Figure S1. Frequency dependence of χ' (a) and χ" (c) for **2** at 1.8 K performed under various applied dc fields. (b) Cole-Cole plots obtained using the frequency dependence of χ" for **2** at 1.8 K under various dc field. The solid lines correspond to the best fit obtained with a generalized Debye model. (d) Field dependence of the relaxation time for **2**. The red line represents the fit using Eq. (1).

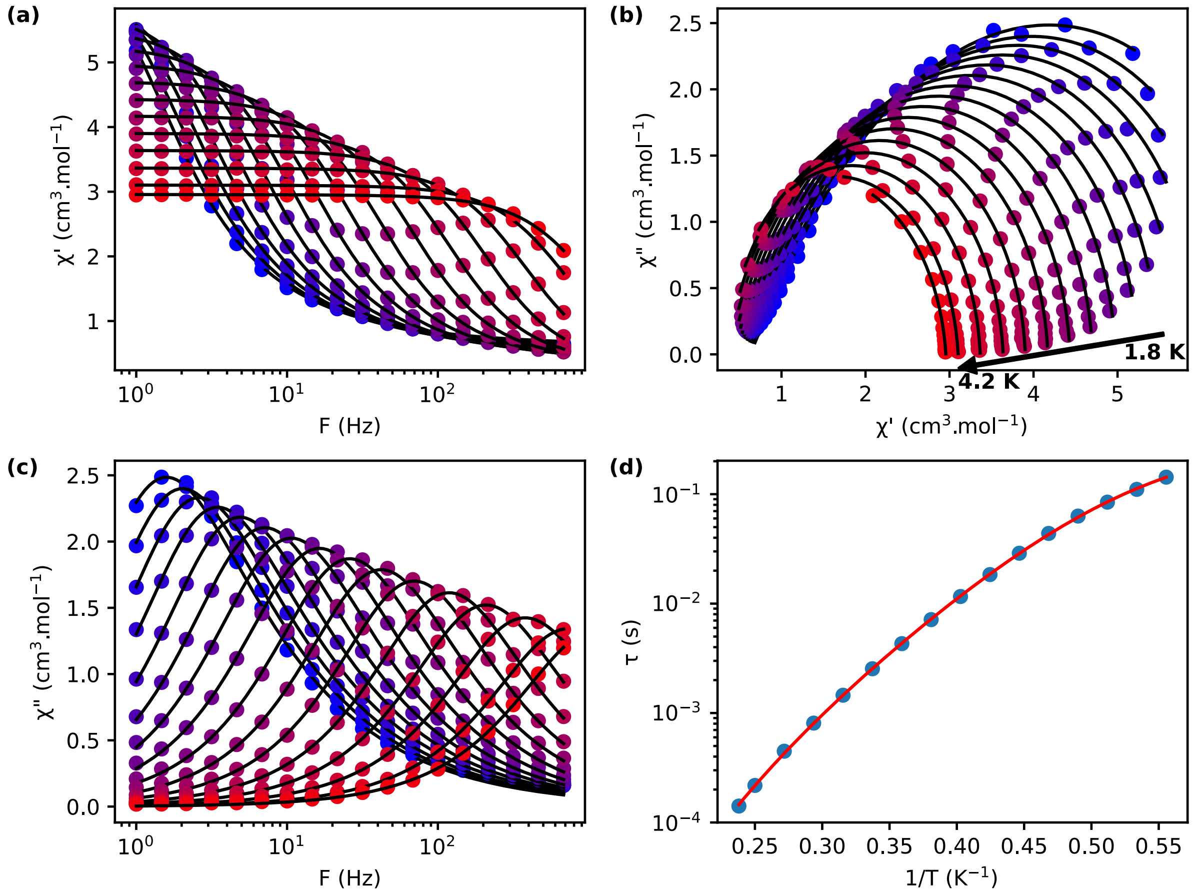

Figure S2. Frequency dependence of the in-phase, χ', (a) and out-of-phase, χ'' (c) components of the ac susceptibility for **2** under optimal applied magnetic field of 500 Oe. The black lines are the result of the Cole-Cole fitting. (b) Cole-Cole plots obtained using the frequency dependence of χ'' for **2** obtained under 500 Oe. The solid lines correspond to the best fit obtained with a generalized Debye model. (d) Temperature dependence of the relaxation time for **2** (500 Oe) and the corresponding fit with Eq. (3) (red solid line).

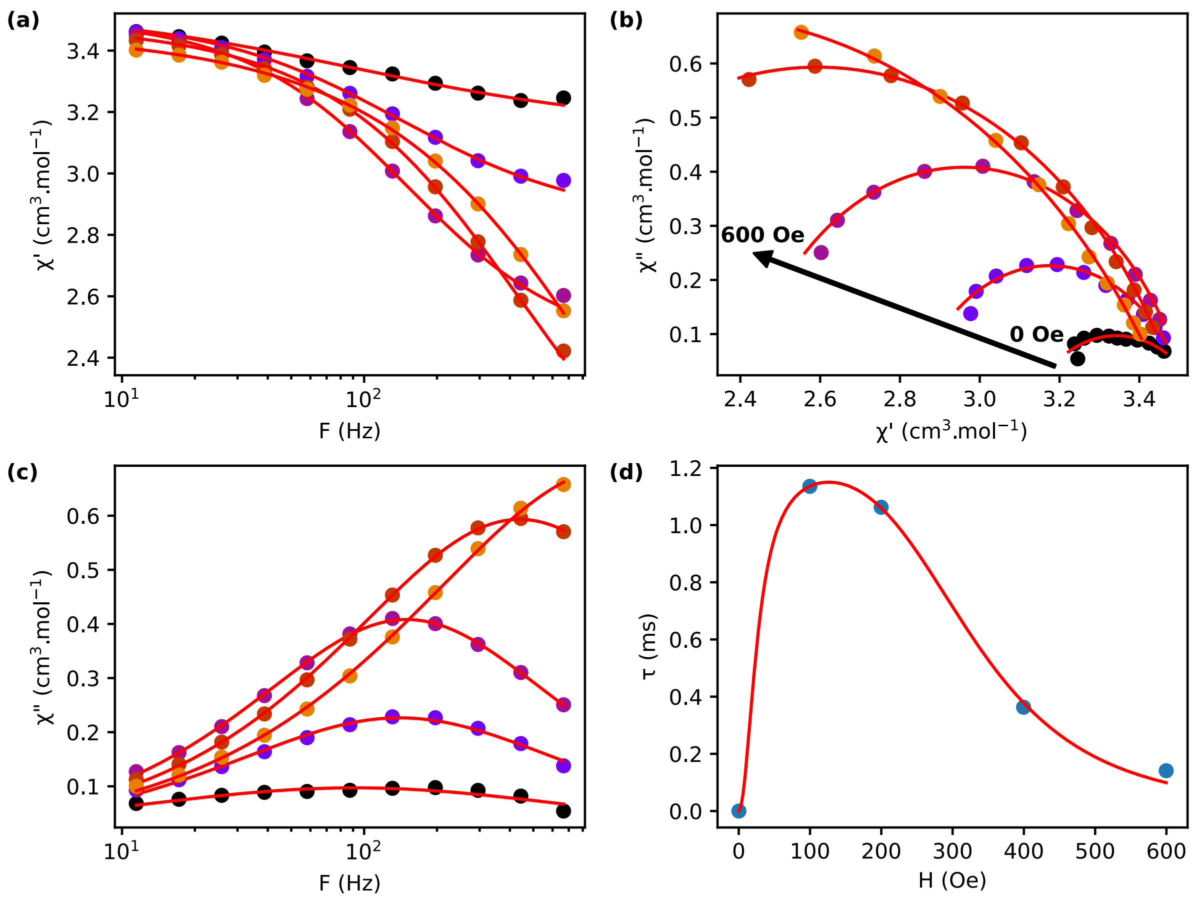

Figure S4. Frequency dependence of χ' (a) and χ'' (c) for **3** at 1.8 K performed under various applied dc fields. (b) Cole-Cole plots obtained using the frequency dependence of χ'' for **3** at 1.8 K under various dc field. The solid lines correspond to the best fit obtained with a generalized Debye model. (d) Field dependence of the relaxation time for **3**. The red line represents the fit using Eq. (1).

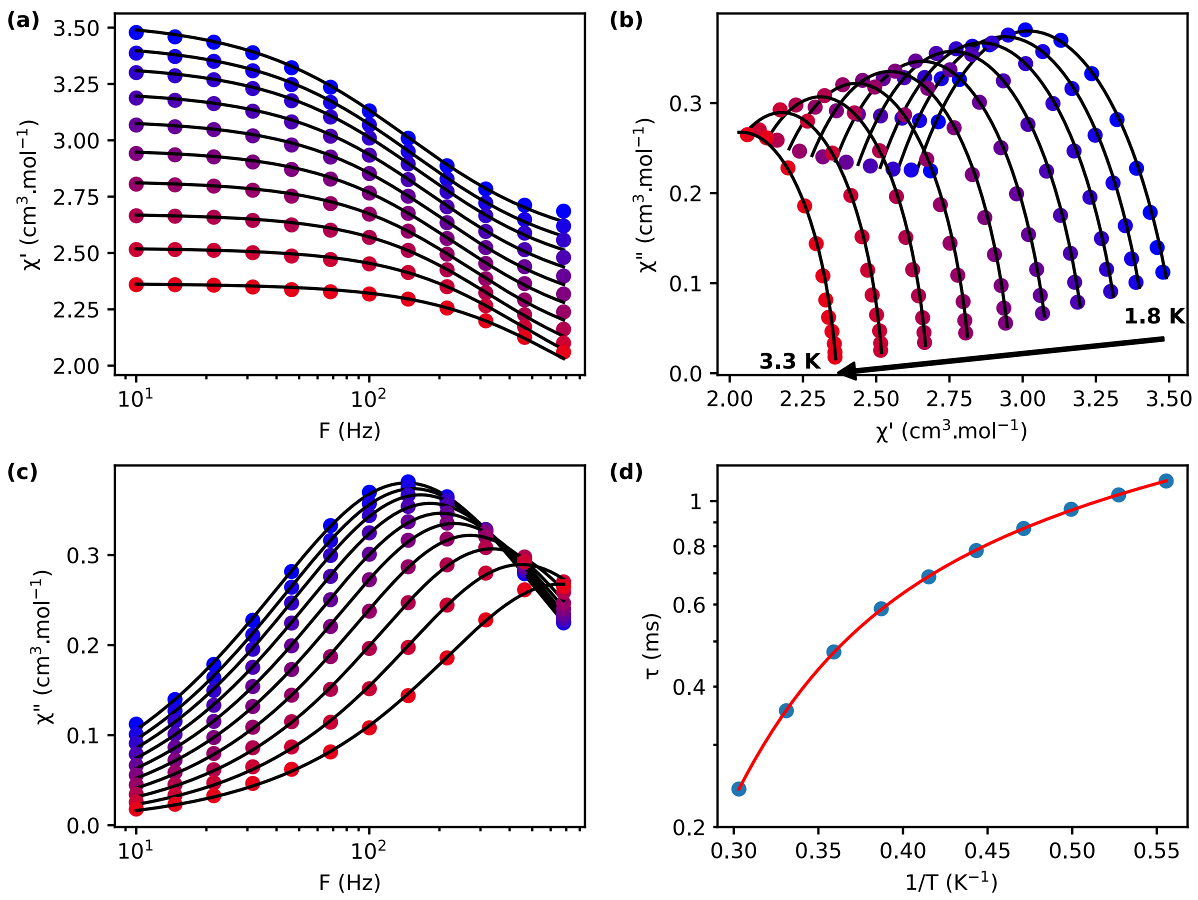

Figure S5. Frequency dependence of the in-phase, χ', (a) and out-of-phase, χ" (c) components of the ac susceptibility for **3** under optimal applied magnetic field of 200 Oe. The black lines are the result of the Cole-Cole fitting. (b) Cole-Cole plots obtained using the frequency dependence of χ" for **3** obtained under 200 Oe. The solid lines correspond to the best fit obtained with a generalized Debye model. (d) Temperature dependence of the relaxation time for **3** (200 Oe) and the corresponding fit with Eq. (3) (red solid line).

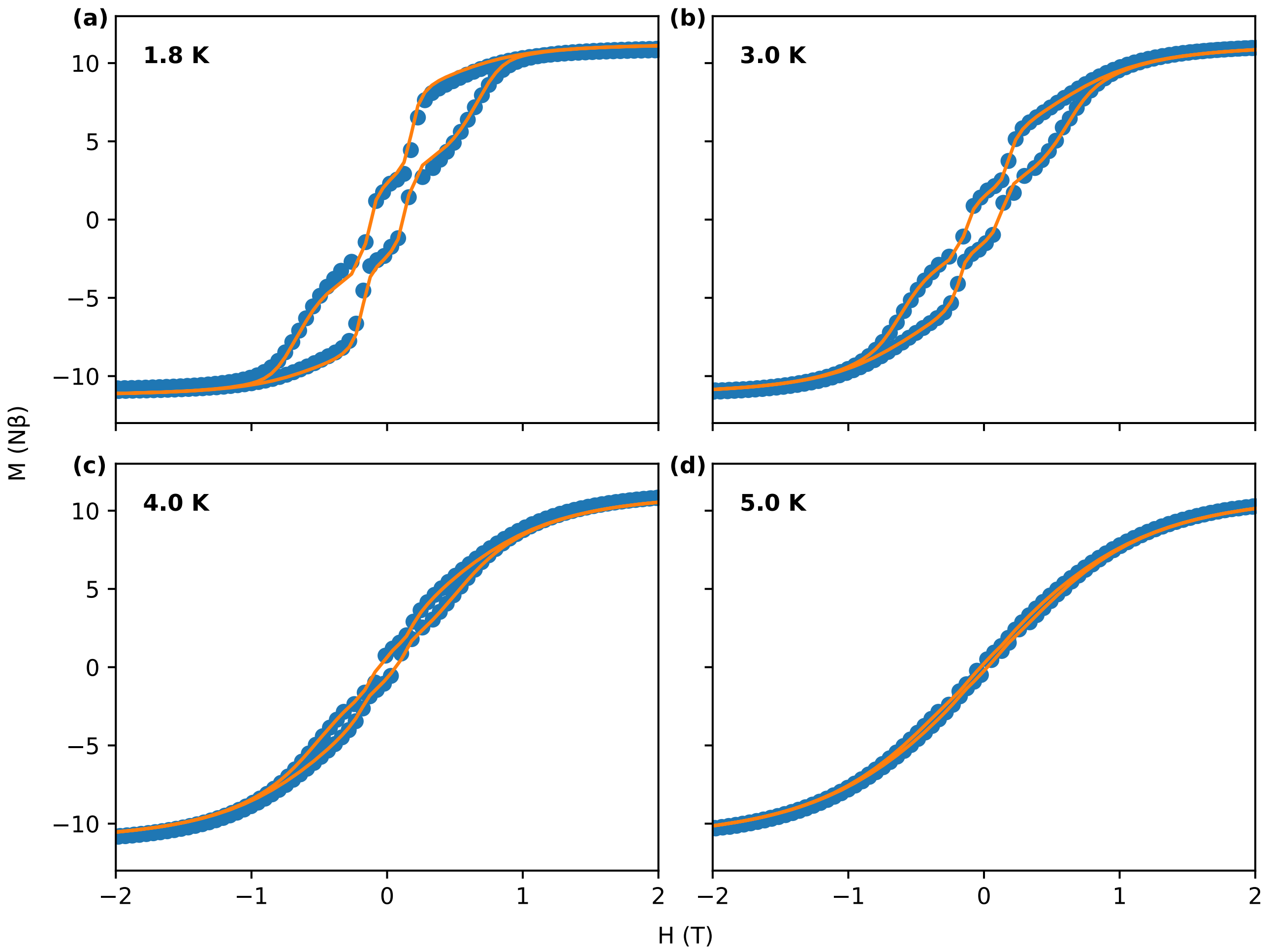

Figure S6. Magnetization as a function of the external magnetic field for sample 4, with a range between -2 Tesla and 2 Tesla and a variation field speed of 100 Oe.s^{-1} at (a) 1.8 K, (b) 3 K, (c) 4 K and (d) 5 K. The plein circles represent the experimental points, and the orange lines represent the theoretical fit.

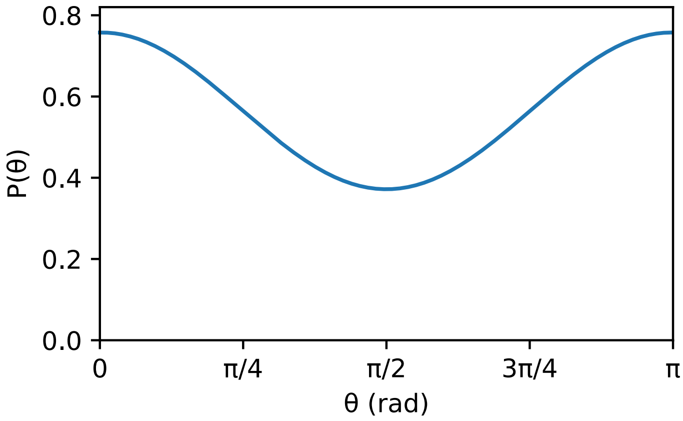

Figure S7. Density of probability of the angle between the magnetic easy axis of the molecules and the external magnetic field as a function of this angle with σ = 1.327 rad.

TABLES

Table S1. Crystal data, data collection and structure refinement details for **1 - 4**.

	**1**	**2**	**3**	**4**
Formula	$C_{54}H_{62}DyO_6$, $C_{24}H_{20}B$, C_7H_9N	$C_{65}H_{61}DyO_5$	$C_{72}H_{60}DyN_3O_3$	$C_{62}H_{78}Cl_6Dy_2Li_2O_8$
M	1395.89	1084.63	1177.73	1502.82
T, K	120	100	120	100
Crystal system	Monoclinic	Monoclinic	Trigonal	Triclinic
Space group	$P2_1/n$	$P2_1/n$	*R3*	*P-1*
Z (*Z'*)	4 (1)	4 (1)	3 (0.333)	1 (0.5)
a, Å	24.162(10)	18.7051(14)	21.568(2)	10.0466(2)
b, Å	12.664(5)	15.0663(11)	21.568(2)	10.8954(2)
c, Å	25.400(10)	20.0426(15)	10.4932(10)	16.9455(3)
α, deg	90	90	90	103.3460(10)
β, deg	114.173(9)	90.639(2)	90	99.6930(10)
γ, deg	90	90	120	109.8920(10)
V, Å^3	7090(5)	5648.0(7)	4227.1(9)	1634.14(5)
d_{calcd}, g·cm^{-3}	1.308	1.276	1.388	1.527
μ, mm^{-1}	11.09	13.7	13.78	25.64
F_{000}	2908	2228	1809	754
$2\theta_{max}$, deg	52	52	50	50
Number of measured refl.	67916	90588	11554	18744
Number of independent refl. (R_{int})	13927	11106	3290	7121
Observed refl. [$I > 2\sigma(I)$]	6880	8823	3285	6930
Parameters	850	648	234	412
R_1 [$F^2 > 2\sigma(F^2)$]	0.0660	0.0595	0.0563	0.0176
wR_2 (all data)	0.1624	0.1589	0.1326	0.0416
$S(F^2)$	0.933	1.106	1.057	1.044
Residual density (d_{max}/d_{min}), e·Å^{-3}	0.868/-1.404	3.229/-2.889	2.183/-0.700	0.947/-0.587

Table S2. Fit parameters for the field dependence of the magnetization performed with Eq. (S1) for compound **4**.

A_{DIRECT} ($s^{-1}.K^{-1}.T^{-4}$)	C_{Temp} (s^{-1}) **1.8 K**	**3.0 K**	**4.0 K**	**5.0 K**	$B_{0,1}$ (s^{-1})	$B_{0,2}$ (T^{-2})	$B_{1,1}$ (s^{-1})	$B_{1,2}$ (T^{-2})	H_{QTM} (T)	J (K)	σ (rad)
0.193 ± 0.002	2.12 ± 0.09	1.63 ± 0.04	0.97 ± 0.03	0.44 ± 0.07	3.89 ± 3.84	(1.5 ± 0.9) 10^8	0.26 ± 0.02s	581 ± 103	0.1611 ± 0.0019	-2.50 ± 0.04	1.327 ± 0.001
