# Supplementary material for: Multifunctional Dy3+ Complexes with Triphenylmethanolates: Structural Diversity, Luminescence, and Magnetic Relaxation
Source: Molecules. 2024 Nov 13;29(22):5343. doi: 10.3390/molecules29225343 (PMC11596367; doi:10.3390/molecules29225343)

checkCIF/PLATON report

Structure factors have been supplied for datablock(s) 2

THIS REPORT IS FOR GUIDANCE ONLY. IF USED AS PART OF A REVIEW PROCEDURE FOR PUBLICATION, IT SHOULD NOT REPLACE THE EXPERTISE OF AN EXPERIENCED CRYSTALLOGRAPHIC REFEREE.

No syntax errors found. CIF dictionary Interpreting this report

Datablock: 2

```
Bond precision:   C-C = 0.0115 A          Wavelength=0.71073

Cell:             a=18.7051(14)     b=15.0663(11)      c=20.0426(15)
                  alpha=90          beta=90.639(2)     gamma=90
Temperature:      100 K

                  Calculated                   Reported
Volume            5648.0(7)                    5648.0(7)
Space group       P 21/n                       P 1 21/n 1
Hall group        -P 2yn                       -P 2yn
Moiety formula    C65 H61 Dy O5 [+ solvent]    C65 H61 Dy O5
Sum formula       C65 H61 Dy O5 [+ solvent]    C65 H61 Dy O5
Mr                1084.64                      1084.63
Dx,g cm-3         1.276                        1.276
Z                 4                            4
Mu (mm-1)         1.370                        1.370
F000              2228.0                       2228.0
F000'             2228.03
h,k,lmax          23,18,24                     23,18,24
Nref              11114                        11106
Tmin,Tmax         0.781,0.814                  0.064,0.100
Tmin'             0.760

Correction method= # Reported T Limits: Tmin=0.064 Tmax=0.100
AbsCorr = MULTI-SCAN

Data completeness= 0.999            Theta(max)= 26.000

R(reflections)= 0.0595( 8823)                         wR2(reflections)=
                                                      0.1586( 11106)
S = 1.104                   Npar= 648
```

The following ALERTS were generated. Each ALERT has the format
test-name_ALERT_alert-type_alert-level.
Click on the hyperlinks for more details of the test.

Alert level A

```
PLAT971_ALERT_2_A Check Calcd Resid. Dens.  1.07Ang From Dy1            3.54 eA-3
```

Author Response: The best available crystals were twinned thin needles suffering from a disorder.

Alert level B

```
PLAT971_ALERT_2_B Check Calcd Resid. Dens.  1.18Ang From O3'            2.51 eA-3
```

Author Response: The best available crystals were twinned thin needles suffering from a disorder.

```
PLAT972_ALERT_2_B Check Calcd Resid. Dens.  1.61Ang From C33           -3.44 eA-3
```

Author Response: The best available crystals were twinned thin needles suffering from a disorder.

Alert level C

```
PLAT241_ALERT_2_C High   'MainMol' Ueq as Compared to Neighbors of        C7S Check
PLAT342_ALERT_3_C Low Bond Precision on  C-C Bonds ...............    0.01152 Ang.
PLAT911_ALERT_3_C Missing FCF Refl Between Thmin & STh/L=    0.600          7 Report
                1  0  1,  13  1  1,  -9  0  5,  -8  0  8,  -6  0  8,  -7  1  9,
               -3  2 11,
PLAT918_ALERT_3_C Reflection(s) with I(obs) much Smaller I(calc) .          1 Check
PLAT971_ALERT_2_C Check Calcd Resid. Dens.  0.39Ang From O1'            2.50 eA-3
```

Author Response: The best available crystals were twinned thin needles suffering from a disorder.

```
PLAT971_ALERT_2_C Check Calcd Resid. Dens.  0.45Ang From O3'            2.26 eA-3
```

Author Response: The best available crystals were twinned thin needles suffering from a disorder.

```
PLAT971_ALERT_2_C Check Calcd Resid. Dens.  1.35Ang From C1             2.04 eA-3
```

Author Response: The best available crystals were twinned thin needles suffering from a disorder.

```
PLAT971_ALERT_2_C Check Calcd Resid. Dens.  1.15Ang From C34           1.81 eA-3
```

Author Response: The best available crystals were twinned thin needles suffering from a disorder.

```
PLAT971_ALERT_2_C Check Calcd Resid. Dens.  0.82Ang From C33           1.77 eA-3
```

Author Response: The best available crystals were twinned thin needles suffering from a disorder.

```
PLAT977_ALERT_2_C Check Negative Difference Density on H7SA      .      -0.46 eA-3
```

Alert level G

```
PLAT002_ALERT_2_G Number of Distance or Angle Restraints on AtSite          6 Note
PLAT083_ALERT_2_G SHELXL Second Parameter in WGHT  Unusually Large      62.69 Why ?
PLAT171_ALERT_4_G The CIF-Embedded .res File Contains EADP Records          3 Report
PLAT176_ALERT_4_G The CIF-Embedded .res File Contains SADI Records          2 Report
PLAT301_ALERT_3_G Main Residue  Disorder .............(Resd    1)          3% Note
PLAT605_ALERT_4_G Largest Solvent Accessible VOID in the Structure        186 A**3
PLAT720_ALERT_4_G Number of Unusual/Non-Standard Labels ..........         16 Note
              H1SA    H1SB    H3SA    H3SB    H4SA    H4SB    H5SA    H5SB
              H6SA    H6SB    H7SA    H7SB    H8SA    H8SB    H9SA    H9SB
PLAT860_ALERT_3_G Number of Least-Squares Restraints .............          2 Note
PLAT868_ALERT_4_G ALERTS Due to the Use of _smtbx_masks Suppressed          ! Info
PLAT883_ALERT_1_G No Info/Value for _atom_sites_solution_primary .     Please Do !
PLAT930_ALERT_2_G FCF-based   Twin Law ( 1 0 1)         Est.d BASF       0.12 Check
PLAT931_ALERT_5_G CIFcalcFCF  Twin Law ( 1 0 1)         Est.d BASF       0.13 Check
PLAT933_ALERT_2_G Number of HKL-OMIT Records in Embedded .res File          3 Note
               -9  0  5,  -6  0  8,  -3  2 11,
PLAT960_ALERT_3_G Number of Intensities with I  <   - 2*sig(I) ...          2 Check
PLAT967_ALERT_5_G Note: Two-Theta Cutoff Value in Embedded .res ..       52.0 Degree
PLAT969_ALERT_5_G The 'Henn et al.' R-Factor-gap value ...........      3.632 Note
              Predicted wR2: Based on SigI**2  4.37 or SHELX Weight 14.37
PLAT978_ALERT_2_G Number C-C Bonds with Positive Residual Density.          0 Info
```

```
   1 ALERT level A = Most likely a serious problem - resolve or explain
   2 ALERT level B = A potentially serious problem, consider carefully
  10 ALERT level C = Check. Ensure it is not caused by an omission or oversight
  17 ALERT level G = General information/check it is not something unexpected

   1 ALERT type 1 CIF construction/syntax error, inconsistent or missing data
  15 ALERT type 2 Indicator that the structure model may be wrong or deficient
   6 ALERT type 3 Indicator that the structure quality may be low
   5 ALERT type 4 Improvement, methodology, query or suggestion
   3 ALERT type 5 Informative message, check
```

It is advisable to attempt to resolve as many as possible of the alerts in all categories. Often the minor alerts point to easily fixed oversights, errors and omissions in your CIF or refinement strategy, so attention to these fine details can be worthwhile. In order to resolve some of the more serious problems it may be necessary to carry out additional measurements or structure refinements. However, the purpose of your study may justify the reported deviations and the more serious of these should normally be commented upon in the discussion or experimental section of a paper or in the "special_details" fields of the CIF. checkCIF was carefully designed to identify outliers and unusual parameters, but every test has its limitations and alerts that are not important in a particular case may appear. Conversely, the absence of alerts does not guarantee there are no aspects of the results needing attention. It is up to the individual to critically assess their own results and, if necessary, seek expert advice.

Publication of your CIF in IUCr journals

A basic structural check has been run on your CIF. These basic checks will be run on all CIFs submitted for publication in IUCr journals (*Acta Crystallographica*, *Journal of Applied Crystallography*, *Journal of Synchrotron Radiation*); however, if you intend to submit to *Acta Crystallographica Section C* or *E* or *IUCrData*, you should make sure that full publication checks are run on the final version of your CIF prior to submission.

Publication of your CIF in other journals

Please refer to the *Notes for Authors* of the relevant journal for any special instructions relating to CIF submission.

PLATON version of 22/08/2024; check.def file version of 21/08/2024

Datablock 2 - ellipsoid plot

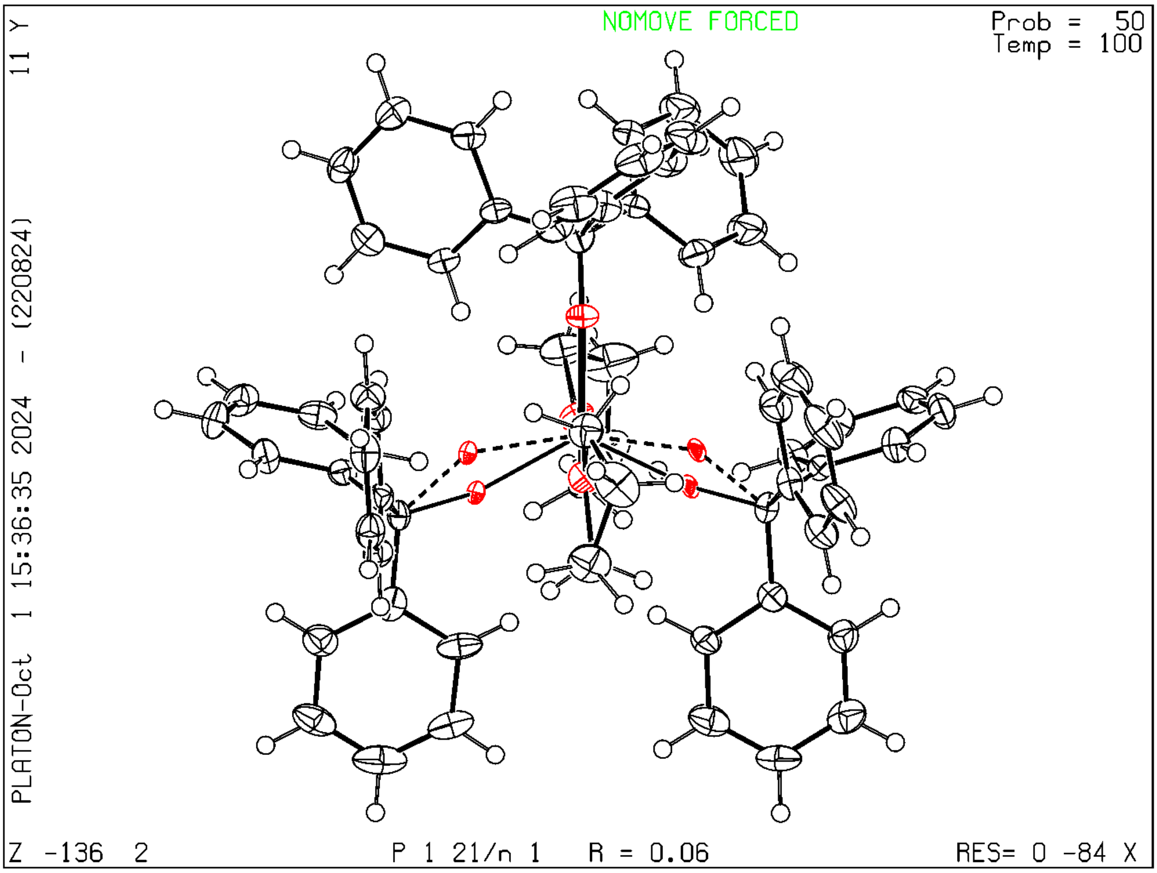

Supplement: Supplementary file 1 [file checkcif_2.pdf › checkcif_2.pdf]
