# Supplementary material for: Multifunctional Dy3+ Complexes with Triphenylmethanolates: Structural Diversity, Luminescence, and Magnetic Relaxation
Source: Molecules. 2024 Nov 13;29(22):5343. doi: 10.3390/molecules29225343 (PMC11596367; doi:10.3390/molecules29225343)

checkCIF/PLATON report

Structure factors have been supplied for datablock(s) 3

THIS REPORT IS FOR GUIDANCE ONLY. IF USED AS PART OF A REVIEW PROCEDURE FOR PUBLICATION, IT SHOULD NOT REPLACE THE EXPERTISE OF AN EXPERIENCED CRYSTALLOGRAPHIC REFEREE.

No syntax errors found. CIF dictionary Interpreting this report

Datablock: 3

Bond precision: C-C = 0.0225 A Wavelength=0.71073

Cell:	a=21.568(2)	b=21.568(2)	c=10.4932(10)
	alpha=90	beta=90	gamma=120
Temperature:	120 K		

	Calculated	Reported
Volume	4227.3(9)	4227.1(9)
Space group	R 3	R 3
Hall group	R 3	R 3
Moiety formula	C72 H60 Dy N3 O3	C72 H60 Dy1 N3 O3
Sum formula	C72 H60 Dy N3 O3	C72 H60 Dy N3 O3
Mr	1177.73	1177.73
Dx,g cm-3	1.388	1.388
Z	3	3
Mu (mm-1)	1.378	1.378
F000	1809.0	1809.0
F000'	1809.02	
h,k,lmax	25,25,12	25,25,12
Nref	3312[1656]	3290
Tmin,Tmax	0.984,0.986	0.017,0.051
Tmin'	0.759	

Correction method= # Reported T Limits: Tmin=0.017 Tmax=0.051
AbsCorr = MULTI-SCAN

Data completeness= 1.99/0.99 Theta(max)= 24.972

R(reflections)= 0.0563(3285) wR2(reflections)= 0.1326(3290)

S = 1.057 Npar= 234

The following ALERTS were generated. Each ALERT has the format
test-name_ALERT_alert-type_alert-level.
Click on the hyperlinks for more details of the test.

Alert level A

```
PLAT973_ALERT_2_A Check Calcd Positive Resid. Density on       Dy1       2.18 eA-3
```

Author Response: The best available crystals were thin twinned needles suffering from a disorder.

Alert level B

```
PLAT342_ALERT_3_B Low Bond Precision on  C-C Bonds ...............     0.0225 Ang.
```

Author Response: The best available crystals were thin twinned needles suffering from a disorder.

Alert level C

```
RINTA01_ALERT_3_C  The value of Rint is greater than 0.12
            Rint given   0.123
PLAT018_ALERT_1_C _diffrn_measured_fraction_theta_max .NE. *_full          ! Check
PLAT042_ALERT_1_C Calc. and Reported MoietyFormula Strings  Differ     Please Check
              Calc: C72 H60 Dy N3 O3
              Rep.: C72 H60 Dy1 N3 O3
PLAT090_ALERT_3_C Poor Data / Parameter Ratio (Zmax > 18) ........       7.08 Note
PLAT094_ALERT_2_C Ratio of Maximum / Minimum Residual Density ....       3.12 Report
PLAT975_ALERT_2_C Check Calcd Resid. Dens.  1.04Ang From O1      .       0.53 eA-3
PLAT975_ALERT_2_C Check Calcd Resid. Dens.  0.99Ang From N1      .       0.45 eA-3
```

Alert level G

```
PLAT002_ALERT_2_G Number of Distance or Angle Restraints on AtSite          3 Note
PLAT003_ALERT_2_G Number of Uiso or U(i,j) Restrained non-H Atoms          12 Report
PLAT020_ALERT_3_G The Value of Rint is Greater Than 0.12 .........      0.123 Report
PLAT033_ALERT_4_G Flack x Value Deviates > 3.0 * sigma from Zero .      0.230 Note
PLAT083_ALERT_2_G SHELXL Second Parameter in WGHT  Unusually Large       7.33 Why ?
PLAT171_ALERT_4_G The CIF-Embedded .res File Contains EADP Records          6 Report
PLAT176_ALERT_4_G The CIF-Embedded .res File Contains SADI Records          1 Report
PLAT186_ALERT_4_G The CIF-Embedded .res File Contains ISOR Records          1 Report
PLAT301_ALERT_3_G Main Residue  Disorder .............(Resd    1)         23% Note
PLAT860_ALERT_3_G Number of Least-Squares Restraints .............         74 Note
PLAT909_ALERT_3_G Percentage of I>2sig(I) Data at Theta(Max) Still       100% Note
PLAT933_ALERT_2_G Number of HKL-OMIT Records in Embedded .res File         10 Note
               11 11  0,  -1  1  1,  -1  5  0, -16 18 -1,   0  8  1,  -3  2  1,
                2 13 -5, -21 20 -2,   5 10 10,   0  3  3,
PLAT961_ALERT_5_G Dataset Contains no Negative Intensities .......     Please Check
PLAT967_ALERT_5_G Note: Two-Theta Cutoff Value in Embedded .res ..       50.0 Degree
PLAT969_ALERT_5_G The 'Henn et al.' R-Factor-gap value ..........      1.217 Note
```

```
            Predicted wR2: Based on SigI**2 10.89 or SHELX Weight 12.55
PLAT978_ALERT_2_G Number C-C Bonds with Positive Residual Density.         0 Info
```

```
   1 ALERT level A = Most likely a serious problem - resolve or explain
   1 ALERT level B = A potentially serious problem, consider carefully
   7 ALERT level C = Check. Ensure it is not caused by an omission or oversight
  16 ALERT level G = General information/check it is not something unexpected

   2 ALERT type 1 CIF construction/syntax error, inconsistent or missing data
   9 ALERT type 2 Indicator that the structure model may be wrong or deficient
   7 ALERT type 3 Indicator that the structure quality may be low
   4 ALERT type 4 Improvement, methodology, query or suggestion
   3 ALERT type 5 Informative message, check
```

It is advisable to attempt to resolve as many as possible of the alerts in all categories. Often the minor alerts point to easily fixed oversights, errors and omissions in your CIF or refinement strategy, so attention to these fine details can be worthwhile. In order to resolve some of the more serious problems it may be necessary to carry out additional measurements or structure refinements. However, the purpose of your study may justify the reported deviations and the more serious of these should normally be commented upon in the discussion or experimental section of a paper or in the "special_details" fields of the CIF. checkCIF was carefully designed to identify outliers and unusual parameters, but every test has its limitations and alerts that are not important in a particular case may appear. Conversely, the absence of alerts does not guarantee there are no aspects of the results needing attention. It is up to the individual to critically assess their own results and, if necessary, seek expert advice.

Publication of your CIF in IUCr journals

A basic structural check has been run on your CIF. These basic checks will be run on all CIFs submitted for publication in IUCr journals (*Acta Crystallographica*, *Journal of Applied Crystallography*, *Journal of Synchrotron Radiation*); however, if you intend to submit to *Acta Crystallographica Section C* or *E* or *IUCrData*, you should make sure that full publication checks are run on the final version of your CIF prior to submission.

Publication of your CIF in other journals

Please refer to the *Notes for Authors* of the relevant journal for any special instructions relating to CIF submission.

PLATON version of 22/08/2024; check.def file version of 21/08/2024

Datablock 3 - ellipsoid plot

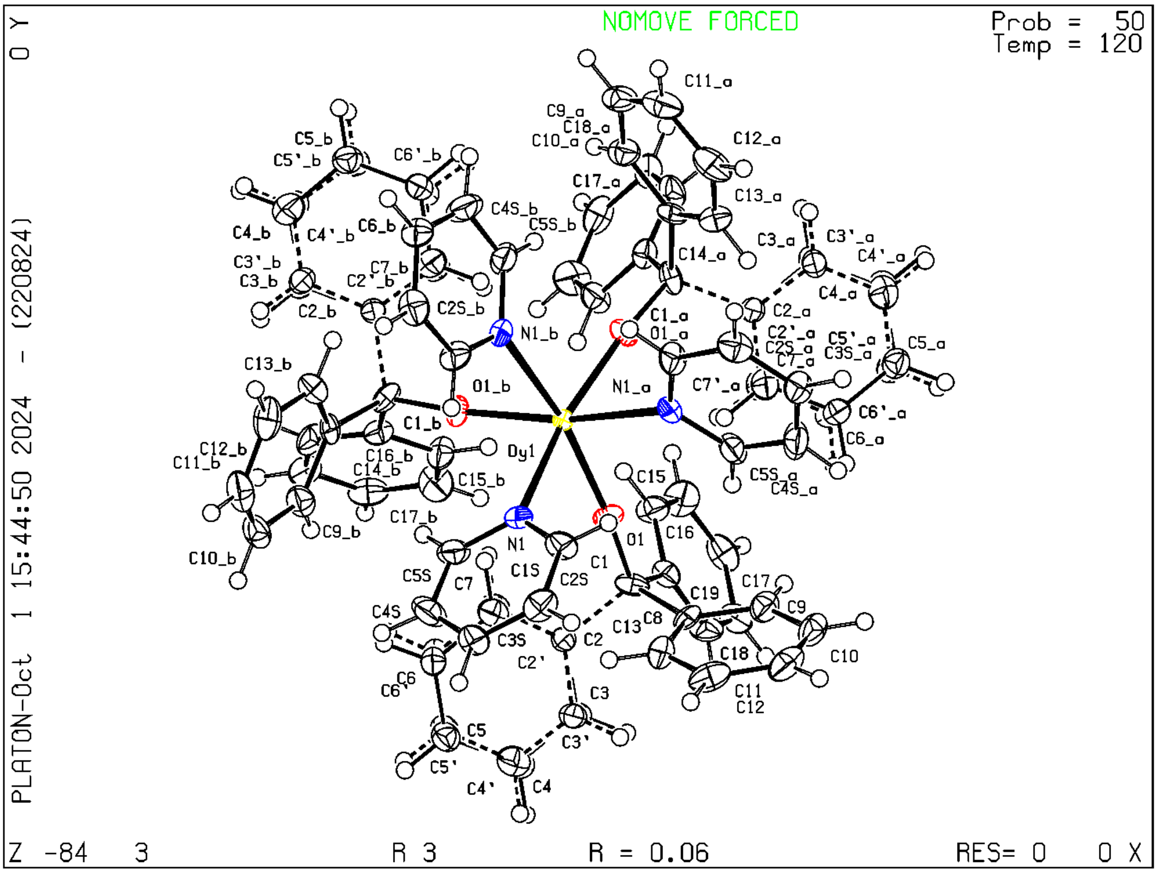

Supplement: Supplementary file 1 [file checkcif_3.pdf › checkcif_3.pdf]
